# Analysis of the Progeny of Sibling Matings Reveals Regulatory Variation Impacting the Transcriptome of Immune Cells in Commercial Chickens

**DOI:** 10.3389/fgene.2019.01032

**Published:** 2019-11-14

**Authors:** Lucy Freem, Kim M. Summers, Almas A. Gheyas, Androniki Psifidi, Kay Boulton, Amanda MacCallum, Rakhi Harne, Jenny O’Dell, Stephen J. Bush, David A. Hume

**Affiliations:** ^1^The Roslin Institute, University of Edinburgh, Edinburgh, United Kingdom; ^2^Mater Research Institute-University of Queensland, Translational Research Institute, Woolloongabba, QLD, Australia; ^3^Department of Clinical Sciences and Services, Royal Veterinary College, University of London, London, United Kingdom; ^4^Nuffield Department of Clinical Medicine, University of Oxford, Oxford, United Kingdom

**Keywords:** chicken, genome, inbreeding, allele-specific, transcriptome, macrophage

## Abstract

There is increasing recognition that the underlying genetic variation contributing to complex traits influences transcriptional regulation and can be detected at a population level as expression quantitative trait loci. At the level of an individual, allelic variation in transcriptional regulation of individual genes can be detected by measuring allele-specific expression in RNAseq data. We reasoned that extreme variants in gene expression could be identified by analysis of inbred progeny with shared grandparents. Commercial chickens have been intensively selected for production traits. Selection is associated with large blocks of linkage disequilibrium with considerable potential for co-selection of closely linked “hitch-hiker alleles” affecting traits unrelated to the feature being selected, such as immune function, with potential impact on the productivity and welfare of the animals. To test this hypothesis that there is extreme allelic variation in immune-associated genes we sequenced a founder population of commercial broiler and layer birds. These birds clearly segregated genetically based upon breed type. Each genome contained numerous candidate null mutations, protein-coding variants predicted to be deleterious and extensive non-coding polymorphism. We mated selected broiler-layer pairs then generated cohorts of F2 birds by sibling mating of the F1 generation. Despite the predicted prevalence of deleterious coding variation in the genomic sequence of the founders, clear detrimental impacts of inbreeding on survival and post-hatch development were detected in only one F2 sibship of 15. There was no effect on circulating leukocyte populations in hatchlings. In selected F2 sibships we performed RNAseq analysis of the spleen and isolated bone marrow-derived macrophages (with and without lipopolysaccharide stimulation). The results confirm the predicted emergence of very large differences in expression of individual genes and sets of genes. Network analysis of the results identified clusters of co-expressed genes that vary between individuals and suggested the existence of trans-acting variation in the expression in macrophages of the interferon response factor family that distinguishes the parental broiler and layer birds and influences the global response to lipopolysaccharide. This study shows that the impact of inbreeding on immune cell gene expression can be substantial at the transcriptional level, and potentially opens a route to accelerate selection using specific alleles known to be associated with desirable expression levels.

## Introduction

A large proportion of causal genetic variation implicated in complex traits in humans is associated with regulatory variants that impact on the level of gene expression (Zhu et al., 2016). Gene expression is itself a complex trait, controlled by both *cis*-acting and *trans*-acting (epistatic) variants and interactions with environment (GTEx project, 2017). Gene expression can therefore provide an intermediate phenotype in analysis of complex traits, an approach that has been termed genetical genomics [(Johnsson et al., 2018) and references therein]. At a population level, expression quantitative trait loci (eQTL) studies of individual cells or tissues can reveal associations between single nucleotide variants (SNVs) and the amount of each mRNA transcribed from the genome (GTEx project, 2017). At the level of an individual, provided there are expressed SNVs, RNA sequencing enables the identification of regulatory variation within a locus, based upon the relative expression of the two alleles (so-called allele-specific expression, ASE) (Pastinen, 2010).

Modern western broiler and layer chickens have been divergently selected for meat and egg production respectively, increasingly using genomic selection with dense genotyping chips (Kranis et al., 2013; Gheyas et al., 2015). This intense selection has generated selective sweeps around regions associated with production traits, for example genes linked to appetite, growth, metabolic regulation and carcase traits in broilers and egg-production in layers (Qanbari et al., 2019). Analysis of linkage disequilibrium in commercial broiler, white egg and brown egg layers revealed highly divergent patterns between selected populations, significant inbreeding coefficients and, on average, much larger average LD blocks than in human populations (Pengelly et al., 2016). The selection for production traits could potentially impact inadvertently (through co-selection) or directly on the immune system which makes an important contribution to fitness in production animals. In the current study we take a novel approach to identifying the potential immunological consequences of trait selection in commercial chickens.

Macrophages are an essential component of the innate immune system. In mammals, the proliferation and differentiation of macrophages depends upon signaling through the macrophage colony-stimulating factor receptor (CSF1R) *via* two ligands, CSF1 and interleukin 34 (IL34). This system is functionally conserved in birds (Garceau et al., 2010). Recombinant CSF1 can be used to generate pure populations of macrophages *in vitro* from bone marrow progenitors (Garceau et al., 2010). We used this system to demonstrate that genes on the Z chromosome in birds are generally not fully dosage compensated in male (ZZ) versus female (ZW) birds. We showed also that the presence of the interferon genes on the Z chromosome impacts on the relative response of male and female macrophages to bacterial lipopolysaccharide (LPS) (Garcia-Morales et al., 2015). To analyze chicken macrophage biology *in vivo* we have produced *CSF1R* reporter transgenic lines on a conventional layer genetic background (Balic et al., 2014; Garceau et al., 2015).

There is a strong signature of selection over the *CSF1R* locus in commercial broilers (Stainton et al., 2017). Analysis of the genomic sequence data for commercial birds (Gheyas et al., 2015) revealed high prevalence non-synonymous protein-coding variants in *CSF1R* that are unique to either broilers or layers (Hume et al., 2019). In support of the possibility that this variation is functionally significant, mutations in either *Csf1r* or *Csf1* in both mice and rats produce severe post-natal growth retardation (Dai et al., 2002; Pridans et al., 2018). Such variation could obviously also impact on innate immune function. Chicken meat and egg production at scale generally involves housing in well-controlled environments and infection control with vaccines and/or prophylactic antibiotics. These production systems may mask the impact of selection on immune-related traits. Increasingly, the efficacy of vaccines is challenged by pathogen evolution and antibiotic use is now largely prohibited. There has therefore been a renewed interest in breeding for disease resistance and in the identification of markers of disease severity and prognosis. One novel strategy for improving disease resistance is based upon selective breeding of birds that display high levels of inducible pro-inflammatory cytokines (IL6 or the CXCL chemokines) in response to bacterial stimuli (Swaggerty et al., 2008; Swaggerty et al., 2016; Swaggerty et al., 2017; Swaggerty et al., 2019).

Most potential regulatory and protein-coding variants of large effect in commercial birds are masked because of the breeding pyramid approach used. Independent pedigree lines are intensively selected for specific traits and then crossed to maximize heterozygosity in the production animals which may contain genetic contributions from as many as eight heavily-selected founder lines. One presumption in such breeding pyramids is that maximal heterozygosity conceals potentially deleterious alleles; leading to hybrid vigor or heterosis. The reciprocal of heterosis is the well-documented phenomenon of inbreeding depression (Charlesworth and Willis, 2009; Chen, 2013). The molecular basis for both phenomena has been studied more extensively in plants than in animals. At least some of the variation underlying heterosis is regulatory and can be detected at the level of mRNA expression of individual genes, where the level of expression in an F1 hybrid commonly lies at the midpoint of expression of parental lines. RNAseq analysis has been used to address this prediction in a defined cross of two inbred chicken lines, in which gene expression was compared in the brain and liver of the parents and embryonic F1 birds. This approach provided strong evidence of frequent allelic imbalance in embryonic brain and liver. In the large majority of cases the combined expression of individual transcripts from the two parental alleles in the F1 animals was essentially additive (Zhuo et al., 2017; Zhuo et al., 2019). A small subset of transcripts showed evidence of dominance or over-dominance in expression level; these were identified as candidate *trans*-regulators contributing to heterosis. The key conclusion from these studies is that the *cis*-acting regulation in the parent lines was demonstrable and heritable in the F1 progeny (as allele-specific expression).

The identification of functional allelic variants based upon RNAseq depends upon the presence of informative expressed SNVs in each individual. An alternative approach that is practical in birds, which are multiparous, is to brother-sister mate F1 progeny from a defined parental cross to generate an F2 population in which the grandparental allelic variants at each locus will be homozygous in a subset of birds. Based upon the results from the inbred cross (Zhuo et al., 2017; Zhuo et al., 2019) such a breeding strategy should expose high and low expression alleles that are masked in heterozygotes. Because of their genetic divergence, broiler-layer intercrosses have been used extensively in QTL mapping of production traits [e.g. (Campos et al., 2009; Hocking et al., 2012; Podisi et al., 2013)]. Aside from their regulated expression of genes encoding immunological functions, macrophages also express a very large proportion of the entire transcriptome at detectable levels (Bush et al., 2018). Given their complex transcriptome and the regulatory functions of macrophages in growth and development, it is conceivable genetic variants that control expression of genes involved in the production traits that distinguish broilers and layers also regulate their expression in macrophages or other cells of the immune system. Therefore, to explore these concepts, we have generated a series of families of F2 individuals from sibling matings derived from a cross between commercial broilers and our *CSF1R*-mApple transgenic line (Balic et al., 2014) which is maintained on an outbred layer background and which expresses the mApple reporter in cells of macrophage lineage. Our analysis of expression variance in immune cells in these inbred birds supports the existence of strong allele-specific expression variants in the parental commercial birds.

## Materials and Methods

### Ethical Approval

All animal work including breeding and care was conducted in accordance with guidelines of the Roslin Institute and the University of Edinburgh and carried out under the regulations of the Animals (Scientific Procedures) Act 1986 under Home Office project license PPL 60/4420. Approval was obtained from the Protocols and Ethics Committees of the Roslin Institute and the University of Edinburgh.

### Animals

Commercial Ross 308 broilers as founders were obtained as hatchlings from PD Hook (Hatcheries) Ltd, Cote, Brampton, Oxfordshire, UK. Founders from the *CSF1R*-mApple reporter transgenic layer line on an ISA-Brown genetic background (Balic et al., 2014) were produced in The Roslin Institute. All birds were bred and housed in approved facilities within the National Avian Research Facility at The Roslin Institute.

### Cell Culture and mRNA Isolation

Bone marrow cells from the femurs of adult or hatchling birds were harvested and cultured for 7 days in recombinant chicken CSF1 to generate a population of bone marrow-derived macrophages (BMDM) as described previously (Garceau et al., 2015; Garcia-Morales et al., 2015; Bush et al., 2018). The macrophages were detached from the plates and re-seeded in 6 well plates with CSF1, with or without 100 ng/ml of LPS, for 24h prior to harvest and purification of mRNA (Garcia-Morales et al., 2015). Spleens were obtained immediately after euthanasia, snap frozen in entirety and stored in RNA-later at −80°C until used for RNA extraction.

### Genetic Analysis

Whole genome sequencing of DNA from the set of founder broiler and transgenic layer lines was performed by Edinburgh Genomics, University of Edinburgh, UK, using the Illumina HiSeqX platform. Sample specific libraries were created from genomic DNA using Illumina SeqLab specific TruSeq Nano High Throughput library preparation kits in conjunction with the Hamilton MicroLab STAR and Clarity LIMS X Edition. The gDNA samples were normalized to the concentration and volume required for the Illumina TruSeq Nano library preparation kits, then sheared to a 450 bp mean insert size. The inserts were ligated with blunt ended, A-tailed, size selected, TruSeq adapters and enriched using eight cycles of PCR amplification. The libraries were normalized, denatured, and pooled in eights for clustering and sequencing. The number of paired reads varied from 177,300,445 to 365,616,593, with an average of 265 million paired reads per sample equating to >30× coverage. The read length varied from 35 bases to 150 bases, with modal read length being 150 bases. Sequence quality was checked with FASTQC (v0.11.8) package. Poor quality bases from the ends of reads were trimmed with Trimmomatic v3.5 software. Reads were trimmed with criteria:

- Remove leading low quality (Phred <20) or N bases (LEADING:20).
- Scan the read with a 10-base wide sliding window, cutting when the average quality per base within a window drops below 20 (SLIDINGWINDOW:10:20).
- After the above operations, drop reads less than 50 bp long (MINLEN:50).

The trimming step retained 54% to 85% of the reads and the trimmed read lengths varied between 50 to 150 bp, with the vast majority of reads between 140–150 bp long. This resulted in overall coverage ranging from 30× to 70×.

Sequence reads from each sample were mapped against chicken reference genome (Galgal5.0) using BWA (v 0.7.8) with BWA_MEM algorithm. The resultant bam files were further processed to mark duplicate reads using Picard tools (v2.1.1) followed by indel realignment using GATK (v 3.7.0). In order to make sure that no contamination or mislabeling occurred during the sample/data processing steps, we checked the realigned bam files for the presence and absence of HIV1 (GAGAGAGATGGGTGCGAGAG) and HIV2 (GCTGTGCGGTGGTCTTACTT) primer sequences that flank the MacApple transgene. As expected, these primer sequences were present only in the transgenic layer birds while absent in the non-transgenic broiler samples.

Variant calling was initially performed with SAMtools mpileup (v 1.1) in conjunction with bcftools with minimum base and mapping qualities set as 20. The variants were annotated against the genomic features annotated by NCBI using package snpEff (v 4.2). Nonsynonymous SNPs were further predicted for their effects on protein sequence changes using the SIFT algorithm in Variant Effect Predictor (VEP). All the variants were also checked for their overlap with evolutionary constraint elements detected from multiple alignments of 49 bird species (https://pag.confex.com/pag/xxiv/webprogram/Paper21473.html) using bedtools (v 2.22.1).

For the selection of F1 breeding pairs, the genotyping of variants within selected genes (*IL10, CSF1R, IL12B, IL34*) used a custom KASP™ (competitive allele-specific PCR) analysis (Biosearch Technologies, Teddington, UK). SNPs from these genes were chosen based on contrasting genotypes and allele frequency in broiler and layers, and annotation results to include potentially functional variants.

### Gene Expression Analysis

Library preparation for RNA sequencing (RNAseq) was also performed by Edinburgh Genomics using the Illumina TruSeq mRNA (poly-A selected) library preparation protocol. mRNA was sequenced at a depth of >40 million strand-specific 75 bp paired end reads per sample, using an Illumina HiSeq 4000. Expression was quantified using the high speed quantification tool Kallisto v0.43.1 (Bray et al., 2016) following procedures detailed previously (Bush et al., 2017; Bush et al., 2018). Kallisto quantifies expression at the transcript level by building an index of k-mers from a set of reference transcripts and then mapping the RNA-seq reads to it, matching k-mers generated from the reads with the k-mers present in the index. Transcript-level estimates (transcripts per million, TPM) are then summarised to the gene level. The current analysis used the revised chicken reference transcriptome defined previously (Bush et al., 2018).

### Network Analysis

Network analysis was performed using Graphia (Kajeka Ltd, Edinburgh, UK). This software builds a correlation matrix based on gene expression patterns, either for sample-to-sample or gene-to-gene comparisons. A network graph was constructed for all relationships above a threshold Pearson correlation coefficient (as detailed in *Results*), connecting nodes (genes) by edges (correlations between nodes above the threshold). Clustering of nodes within the network was performed for the gene-to-gene analysis using the Markov clustering (MCL) algorithm at an inflation value of 1.7 to decrease granularity resulting from the similarity of samples all from the same cell type. Functional annotation of clusters of genes used DAVID 6.8 (https://david.ncifcrf.gov/home.jsp). 

### Analysis of Gene Expression in a Low Fitness Family

The expression level of genes in the BMDM samples was averaged over all samples in one family (Family H; N = 6) in which there was poor hatch rate and low body weight and all other samples (N = 22 untreated and N = 25 LPS treated). All genes where the value of at least one of these averages was greater than 1 were included in the analysis. The ratio of the average in Family H to the average in other samples was calculated. Gene symbols for all samples with a ratio of >1.5 or less than 0.67 (i.e. with at least a 1.5-fold difference) were identified. To identify possible pathways involved in the low fitness, the gene sets were analyzed using the gene ontology analysis software DAVID (see above). and separately in GATHER (https://changlab.uth.tmc.edu/gather/). The analysis was performed for control samples and samples treated with LPS separately.

## Results and Discussion

### Genomic Sequencing

**Figure 1:**
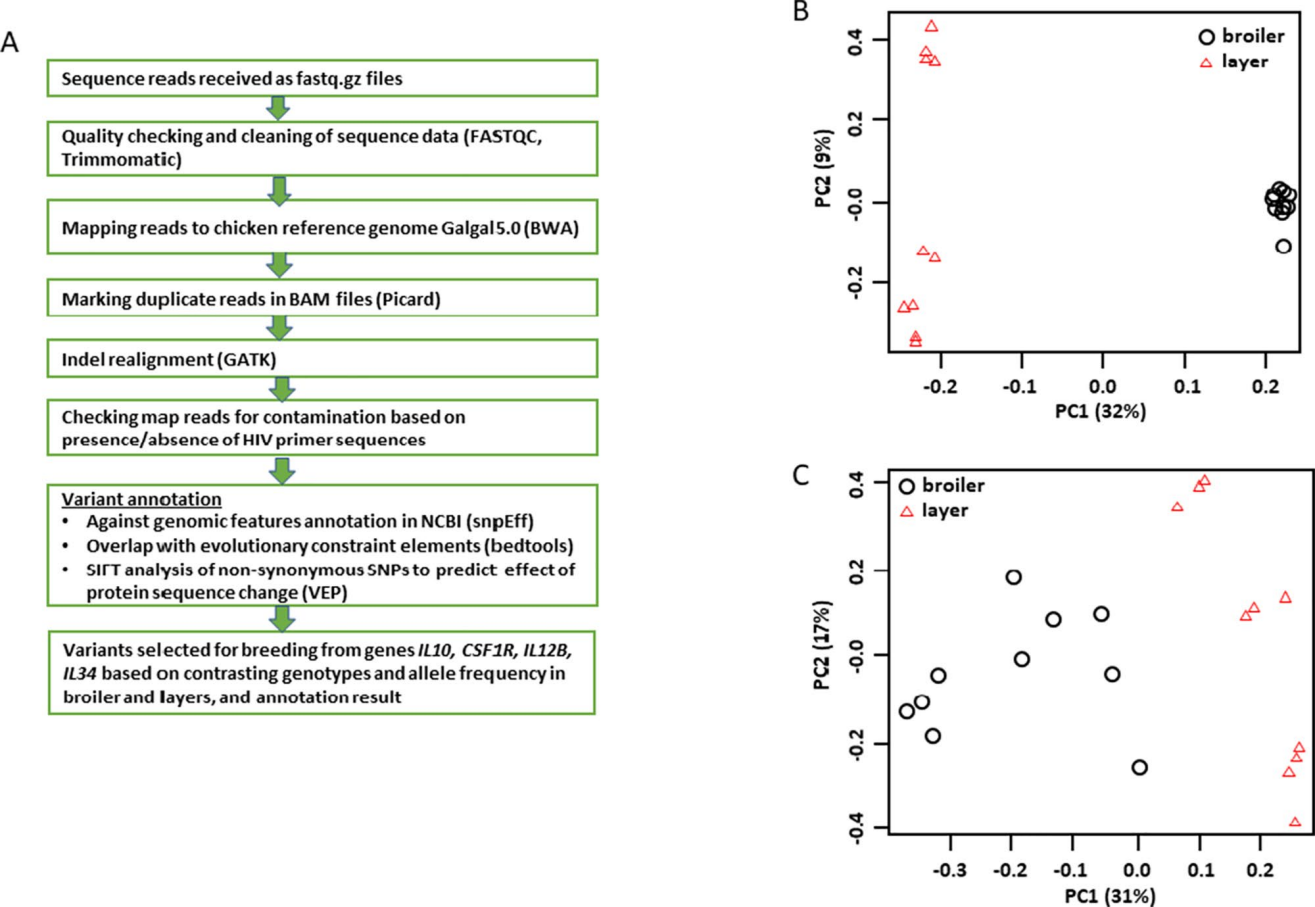
Analysis of genomic sequences of founder layer and broiler birds. Genomic DNA sequences obtained from the 27 commercial broiler and transgenic layer birds used as founders in the inbreeding experiments were analyzed as shown in Panel **(A)**. Panel **(B)** shows a PCA based upon a 13.4M filtered SNV panel derived from the variant call files for the set of birds sequenced. Note the first principle component (PC) separates the layers (left) and the broilers (right). The second PC identifies substantial variation within the two populations with the greatest variation in the layers. Panel **(C)** shows a PCA based solely upon SNVs identified within the members of the IRF gene family (See **Table S6**). Note that PC1 again separates broilers and layers, whereas PC2 identifies substantial variation within breed type.

We completed whole genome sequencing of a total of 10 Ross 308 commercial broilers (five of each sex) and 10 *CSF1R*-mApple transgenic layer birds (five of each sex). **Figure 1A** outlines the analysis and annotation pipeline. Initial variant discovery (with minimum base and mapping quality at 20) indicated a transitions to transversions ratio relative to the reference genome slightly over 2:1 for all samples, which is within normal range. The commercial broiler sequences have a slightly higher level of heterozygosity and much higher numbers of singletons than layers as expected given their derivation from multiple pedigree lines. **Figure 1B** shows a principal component analysis (PCA) of these birds based upon a 13.4 M filtered SNV panel derived from the variant call files for the set of birds sequenced (filtration criteria: min. variant quality 30, min. genotype quality 15, and max. rate of missing genotype 20%). The first principal component clearly separates the broilers from the transgenic layers, whilst the second identifies genetic diversity in the layer population. For reasons that will become evident in subsequent analysis described below, we also generated a PCA based solely upon SNVs detected within loci encoding the members of the interferon-responsive factor (IRF) family, which may produce potential trans-acting transcriptional regulators of inducible gene expression in macrophages. **Figure 1C** shows that the first principal component again separates broilers and layers but the second highlights extensive diversity in both populations.

The main purpose of the whole genome analysis was to identify candidate null protein-coding variants in each of the founder birds and also to determine whether there are prevalent broiler and layer-enriched polymorphic haplotypes in our founders that could be deliberately driven to homozygosity by brother-sister mating. A comparative study of a similar-sized cohort of broiler and layer lines (14 of each) from Brazilian breeds (Boschiero et al., 2018) identified >500 stop gain and >7000 coding variants. Consistent with the genetic diversity identified in this earlier study, the analysis identified a total of 14.1M SNVs in our limited population. The vast majority were non-coding and heterozygous. Within this large set, we identified those predicted to affect exonic sequences, and identified either HIGH impact (i.e. stop gained/lost, start lost, and splice acceptor/donor variants) or nonsynonymous deleterious variants (based on SIFT analysis). We identified 979 SNVs predicted to be HIGH impact and a further 10,872 predicted nonsynonymous coding variants that were annotated as deleterious by SIFT. Often one SNV was associated with more than one predicted impact so it was difficult to place it in a unique group. Amongst the high impact categories that likely produce a complete loss-of-function there were 20 splice_acceptor_variants; 26 splice_donor_variants; 282 start_lost (7 in splice_regions); 628 stop_gained (15 in splice regions); and 142 loss-of-stop codon variants and 1 combined loss-of-start/loss-of-stop. These numbers are approximately consistent with reports of the prevalence of null mutations in chickens based upon much larger cohorts of disparate commercial bird populations (Rubin et al., 2010; Qanbari et al., 2019). Those published studies indicate that few loss-of-function variants are associated with selective sweeps and therefore that such variants are not commonly the direct subject of breeding selection. Indeed, overall amino acid altering mutations were significantly less prevalent in domestic chickens than in their wild red jungle fowl ancestors (Rubin et al., 2010; Qanbari et al., 2019). Nevertheless, there remains very substantial coding sequence polymorphism and potential null mutations in the founder birds that could affect immune and other functions.

Based upon the genome sequencing, we were also able to identify prominent SNVs associated with specific genes of interest that might generate global differences in monocyte-macrophage numbers and/or activation state. **Table 1** summarizes the extensive variation that we identified in the *CSF1R*, *IL34* and *IL10* loci. In addition to *CSF1R* and *IL34* (which could control macrophage differentiation) we focused on *IL10* because of our recent data indicating substantial variance in IL10 production amongst commercial broilers responding to Eimeria parasite challenge (Boulton et al., 2018a; Boulton et al., 2018b) and evidence that endogenous IL10 exerts a feedback regulatory effect of macrophages responding to LPS (Wu et al., 2016). As discussed above, the focus on *CSF1R* and its two ligands was based on their possible roles in growth and selection in broilers. The *CSF1* locus was poorly annotated on the chicken genome at the time breeding decisions were made but it is now clear that this locus lies within 2Mb of *IL10* on chromosome 26. Subsequent analysis on a large population of commercial broilers revealed significant LD between *IL10* and *CSF1* and also very limited heterozygosity at the *CSF1* locus itself (Psifidi A, unpublished). From amongst the candidate SNVs detected in *CSF1R* and *IL34* we did not identify protein-coding variants with predicted large effect, but we did identify SNV markers that were strongly-enriched in either the broiler or layer parents and might potentially be linked to expression variants. In the case of IL34, at position 1780913 on Chr 11, neither broilers, nor layers had the reference allele identified in Red Jungle Fowl, and the two were almost fixed for different alternative alleles (C > T and C > G respectively). Accordingly, these variants were used as markers for inbreeding. Within the *IL10* locus we identified a non-synonymous coding variant (p.Cys4Gly) that was more prevalent in the broiler parents.

**Table 1:** DNA sequence variants in the vicinity of candidate genes of interest detected in 27 founder broiler and layer birds.

Gene	Coordinates in Gal5	No. of Variants	No. of Indels	No. of variants with quality >30	No. HIGH MODERATE effect variants	No. of variants overlapped with constrained elements
CSF1R	Chr 13: 13275507 to 13292566	457	40	453	2	38
CSF1	Chr 26: 1276201 to 1282875	99	21	85	4	0
IL10	Chr 26: 2562275 to 2564509	39	3	38	3	5
IL34	Chr 11: 1776702 to 1781094	98	14	95	1	4

High impact variants include stop gain/loss and splice donor and acceptors. Moderate impact variants includes nonsynonymous SNVs. Constrained elements are evolutionary conserved regions of the chicken genome detected by comparative analysis of 48 bird genomes using GERP++ package (https://pag.confex.com/pag/xxiv/webprogram/Paper21473.html)

Each of the founder birds also carried multiple candidate mutations of large effect. **Table 2** summarizes selected examples of high-confidence protein-coding variants in immune-related genes that in most cases were detected in more than one founder as a heterozygote but were not detected as homozygotes. Amongst these candidates, *TNFRSF10B* and *IL12B* were considered possible regulators that might generate an immune-related phenotype if homozygous for loss-of-function alleles. *TNFRSF10* encodes a protein variously known as TRAIL receptor 2, DR5, and Killer. It is involved in triggering of apoptosis. In humans, there are several *TNFRSF10* genes, but in mice, there is a single *TNFRSF10B* gene and null mutation leads to alterations in radiation-induced cell death (Finnberg et al., 2005). The chicken genome (Galgal5) also has a single *TNFRSF10B* gene.

**Table 2:** Sequence variants affecting genes expressed in immune cells with predicted HIGH effect on protein coding regions.

Gene	Chr	Pos	Ref	Alt	qual	type	Hom alt	Het	Hom ref
TAPBPL	1	76880284	G	T	999	Splicing	0	9	10
HHLA2	1	87500359	CAGAG	CAGAGAAGAG	197	INDEL frameshift	0	1	18
IL18RAP	1	1.34E+08	TTTTTTTTTTTTTTG	T	426	INDEL frameshift	0	9	10
IL8L1	4	51270553	T	A	458	Exonic	0	2	17
TNFSF10	9	19373730	CCTCTC	CCTC	153	INDEL frameshift	0	9	10
TNFRSF10B	22	1281321	C	T	214	Stop gain	0	1	18
IL12B	13	8133187	G	A	999	Exonic	0	5	14
IRF1	13	16983217	GATCCTGTGCTGTGCT	G,GGTGCTGTGCT	960	INDEL frameshift	0	2	9
HMHA1	28	2956121	C	CCA	201	INDEL frameshift	0	2	16
CD69	1	5876	CCAGACAGA	CCAGA	115	INDEL frameshift	0	3	16
CD69	1	5915	GC	G	41	INDEL frameshift	0	3	16
TGFB1	32	27061	CTGGGG	C	416	INDEL	0	2	17
TGFB1	32	28627	CT	C	77	INDEL frameshift	0	7	8
TGFB1	32	28810	CAGCAG	C,CCAG	999	INDEL frameshift	0	14	1

Assuming simple Mendelian inheritance and random mating, around 1/16 birds in the F2 generation will be homozygous for any one of the null mutations present in a heterozygous state in either of its grandparents. In addition to the variants we selected deliberately each F2 bird is likely to be homozygous for a unique subset of the null alleles present in the founder lines. Where the grandparents are homozygous for breed-enriched coding and non-coding allelic variants, all F1 matings are between heterozygotes and 25% of the F2 progeny should be homozygous for any variant if there is no impact on viability.

### Primary Phenotype of F2 Birds

**Figure 2:**
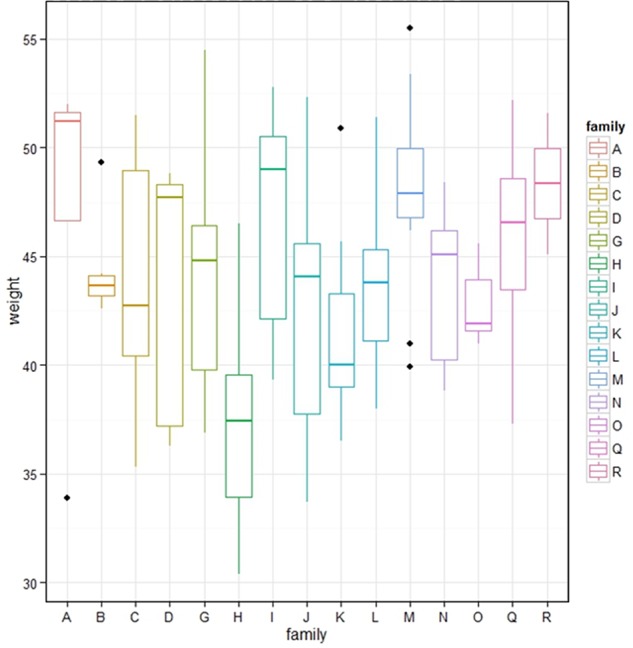
Post hatch body weight in F2 progeny of F1 sibling matings. The figure shows the range of body weights of hatchling birds in each of 15 families derived from sibling mating of the progeny of an F1 broiler-layer cross. Dots identify outliers.

In total, we generated 15 families of F2 progeny from brother-sister matings of F1 birds selected by genotyping for heterozygosity for alleles of interest derived from their founder parents. These birds were also selected to be positive for the *CSF1R*-mApple transgene so that the majority of their offspring could be assessed for transgene expression as a marker. For logistical reasons analysis of F2 phenotypes was carried out exclusively in the immediate 48h post-hatch period. All of the F2 birds were weighed at hatch. **Figure 2** shows box and whisker plots for the distribution of body weights in each F2 family. As expected given the nature of the founder lines, one of which (the broilers) has been selected for rapid body weight gain, there was substantial variation in body weight amongst individual F2 birds even at hatch. In some families there were significant outliers but in only one family, family H, was there a significant reduction in average body weight compared to all other families. In that family, three birds died at or just before hatching with substantial abnormalities including exposed brain, blood in thoracic cavity, muscles and liver, underdeveloped cartilage, and abnormal yolk sac and yolk. The overall hatch rate was also substantially reduced (not shown). For any individual family, 1/4 progeny will be homozygotes for any loss-of-function mutations present in both F1 parents and the number of birds assessed in these families is sufficient to identify outliers or fail-to-hatch numbers. Accordingly, the data indicate that few of the loss-of-function mutations identified in WGS lead to compromised development when bred to homozygosity.

### Monocyte Count

**Figure 3:**
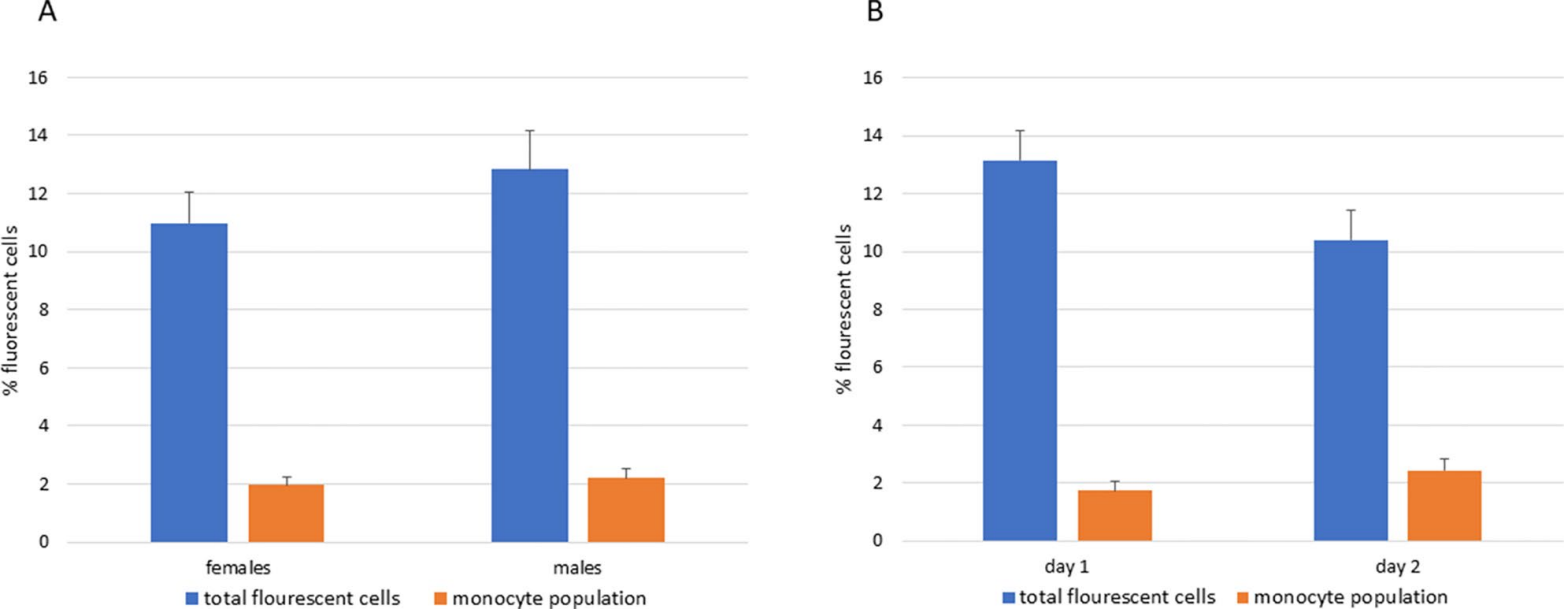
Variation in blood leukocyte populations in F2 progeny of F1 sibling mating. Blood from the entire cohort of hatchling birds was analyzed for total *CSF1R*-mApple reporter gene expression (which measures the myeloid compartment including heterophils) or for blood monocytes (KUL01/mApple+). For logistical reasons these analyses were performed on Day 1 or Day 2 of hatch. Bars show the mean +/- SD. The Figure demonstrates that the variance between birds was small, there was no effect of the sex or day of sampling. **(A)** Blood leukocytes in males and females. **(B)** Blood leukocytes on Day 1 and Day 2 after hatch.

As a preliminary screen for the possible impact of *CSF1*, *IL34*, or *CSF1R* variants we screened all of the F2 progeny by flow cytometry for the number of blood monocytes (co-expressing the surface marker KUL01) and the prevalence of cells expressing the *CSF1R*-mApple reporter gene. The latter percentage is higher because the reporter gene is also expressed in heterophils, albeit at a 10-fold lower level than in monocytes (Balic et al., 2014). When the data from all F2 hatchlings was merged the two parameters were consistent between birds, with no clear outliers, independent of sex (**Figure 3A**) and did not change when measured on day 1 or day 2 post-hatch (**Figure 3B**). With the available sample sizes, we did not detect a significant effect of homozygosity for the parental *CSF1R* or *IL34* allelic variants. Marginal impacts of heterozygous variation in *TNFRSF10B* and homozygosity for *IL10* variation were insignificant when corrected for the number of comparisons. Overall, this analysis did not provide evidence for variants of large effect in the selected genes that distinguish between broilers and layers and which impact upon the blood heterophil or monocyte count in chickens.

### Analysis of Variation of Gene Expression in Spleen of F2 Birds

To screen for variants that influence gene expression in immune cells, we first performed RNAseq on whole spleens from 18 hatchlings derived from F2 families A (1; IL34), C (6; IL12B), G (2; IL10), L (2; IL34), M (3; IL34), Q (3; IL10), R (1; IL10) that segregated the variant alleles for *IL34, IL12B*, and *IL10*. We chose hatchlings to avoid variation that might arise from exposure to infectious agents. Spleen is a mixture of myeloid, lymphoid and other hematopoietic cell populations, each of which has a gene expression signature that can be detected within the total mRNA pool. We profiled males and females from multiple families and individual birds were specifically genotyped for the allelic variants at the *IL34*, *IL12B*, and *IL10* loci to assess whether these variants were associated with any specific pattern of gene expression. SNVs in *IL12B* and *IL10* have been associated with immune-response traits in a large pedigree derived from a white egg/brown egg layer cross (Biscarini et al., 2010) and we considered the possibility that pleiotropic impacts of such genetic variation might manifest in changes in the spleen. In the case of IL34, we reasoned that variation might be associated with monocyte-macrophage number, and hence the relative abundance of macrophage-specific transcripts. For the purpose of the analysis, we removed transcripts that had a maximum expression <10 TPM (see *Methods*). The complete data set from this analysis is provided in **Table S1**.

One consideration in any gene expression analysis in chicken is the difference between males and females. Females are the heterogametic sex and have only one copy of genes on the Z chromosome. As mentioned above, dosage compensation is incomplete in males (the homogametic sex) with two copies of these genes (Garcia-Morales et al., 2015; Zimmer et al., 2016). In **Table S1**, the Z chromosome-specific genes are considered in a separate worksheet. Around 500 transcripts from Z chromosome genes were detected above the expression threshold in macrophages. As reported based upon a smaller set of samples from late-stage embryos, (Zimmer et al., 2016) the large majority of genes on the Z chromosome were expressed more highly in male than in female spleen, although the median ratio was around 1.5-fold rather than twofold suggesting incomplete compensation. Numerous known immune-associated genes (e.g. *CCL19, IL7R, JAK2, CD274, TNFAIP8*), transcription factor genes (*MEF2C, NFIL3*) and metabolic enzyme genes (*ACO1, HMGCR*) on the Z chromosome that were expressed at high levels in spleen appeared to be largely dosage compensated (expression ratio not significantly different from 1). We considered also the possibility that immune-related genes on the Z chromosome might be especially subject to evolutionary selection by virtue of being haploid in females but there was no evidence of variant alleles on the Z chromosome producing more than 2-fold differences in gene expression between F2 individuals. Almost all transcripts varied between individuals across a 2-3 fold range without obvious outliers. The single exception is the antimicrobial gene, avidin, where the level ranged from 5 to 121 TPM.

We next considered the autosomal gene set for spleen. As noted above, these families were created in part based upon the genotype of variants at the *IL34, IL12B* and *IL10* loci. Of these genes, *IL12B* and *IL10* were not expressed above the detection threshold in spleen. *IL34* was expressed, but the level of expression was not correlated with homozygosity for either of the parental SNV alleles. To examine the variability in gene expression, we considered the range of values for individual birds and presented that range as a ratio of maximum to minimum (max/min, giving a fold difference value) (**Table S1**). The max/min ratios for commonly-used reference genes, *ACTB, HPRT* and *GAPDH*, were each around 1.35. The median ratio for the entire data set was 1.6. Of the transcripts for which the max/min ratio was >5, most were known W chromosome-specific transcripts (and others were correlated and likely W chromosome-associated, see below), and the large majority of the remainder had no informative annotation or were annotated specifically as endogenous retroviruses. Recent studies have indicated considerable divergence in endogenous retrovirus insertions in individual birds and provide evidence for their expression in spleen and induction in response to infection (Lee et al., 2017; Qiu et al., 2018; Pettersson and Jern 2019). It is unclear whether the extreme individual variation in expression of these putative retroviral transcripts is functionally important.

To explore whether any of the sets of apparently divergent transcripts were co-regulated, we used the network analysis tool Graphia to generate a sample-to-sample correlation matrix based on expression across the 17 spleen samples, correlated at r ≥ 0.97. This tool has been used previously in the generation of a chicken transcriptional atlas (Bush et al., 2018). There was no association between the samples based on sex of the bird or genotype for any of the genes of interest. We then generated a gene-to-gene correlation matrix. The resulting network graph at a correlation threshold of 0.8 contained 10,273 nodes (genes) connected by 171,821 edges (connections between nodes of r ≥ 0.8). The network was clustered using the MCL algorithm with an inflation value of 1.7. The resulting network graph and the annotated clusters are summarized in **Table S2**. In principle, such an analysis might reveal *trans*-acting variation. For example, over-expression of a growth factor or a transcription factor and its downstream targets would be correlated with each other and cluster together. In the case of the three cytokines of interest, there were no clusters that correlated with homozygosity for any of the allelic variants derived from the grandparents. *IL34* was expressed and varied over a 2.3-fold range but was not correlated with any other transcripts including the receptor gene *CSF1R*.

The clustering revealed a subset of clusters that could be ascribed a biological function, which provides an internal control indicating the power of the approach. Cluster 2 contains the large majority of cell-cycle related genes (including key transcription factors *E2F* and *FOXM1*) identified previously in the chicken transcriptional atlas (Bush et al., 2018). As might be expected, they are highly-expressed in hatchling spleen; the narrow range of average of gene expression values across samples reflects relatively small differences in proliferative cell numbers between the animals. Cluster 4 is made up almost entirely of Z chromosome-associated transcripts, with the average expression around 1.6-fold higher in males than females as discussed above. The small number of transcripts within this cluster that are not Z chromosome-associated are mainly poorly-annotated; only 11 have a current non-Z chromosome assignment. The tight restriction of this cluster to Z chromosome-associated genes indicates that the lack of dosage compensation of genes on the Z chromosome has no downstream impact on expression of transcripts on the autosomes. The reciprocal cluster, Cluster 11, contains W chromosome-specific transcripts that are female-specific in their expression. This cluster also contains multiple candidate protein-coding transcripts that have not been assigned, and only 7 that are currently assigned to an autosome. Cluster 6 is clearly enriched for known markers of lymphocytes (e.g. *CD3E, CD4, CD8A*) and lymphocyte-associated transcription factors (*LEF1, STAT4, TCF7*) and presumably reflects subtle variation in relative lymphocyte content of the spleens. Clusters 10 and 13 contain known epithelial (e.g. *KRT14*) and liver (e.g. *ALB*) associated transcripts respectively, probably reflecting minor contaminants in tissue harvesting. Surprisingly, although macrophages are clearly a major component of the cell populations of the spleen, there was no obvious cluster of macrophage-expressed genes. Macrophage marker genes such as *CSF1R* varied only across a 1.6-fold range but did not cluster with each other. This finding is consistent with the analysis of monocyte numbers above indicating that there is very little inter-individual variation.

Cluster 22 was the only cluster that was both highly-expressed and extremely-variable amongst the samples. It contains several heterophil-specific transcripts encoding granule proteins (*CATHL1, CATHL2, LYSG, MIM1, S100A9*), the cytoplasmic protease inhibitor *SERPINB10* (Rychlik et al., 2014) and the IL8 receptor, *CXCR1*. *CATHL2* is very highly-expressed and anti-CATHL2 antibody has been used a marker for heterophils in hatchling spleen (Cuperus et al., 2016). We conclude that the coordinated variation in this cluster reflects profound variation in heterophil number amongst the different samples. There was no evidence within the cluster of a candidate regulator expressed in spleen that might explain the variation. No candidate regulator was evident even if the correlation threshold was lowered. Genes encoding the CXCR1 ligand (*IL8*) and the G-CSF receptor (*CSF3R*) did vary around 5-fold between samples but were not correlated with each other or with their binding partners. As noted below, we did observe a massive variation in regulated expression of the growth factor gene *CSF3* in macrophages which would provide a clear mechanistic explanation for variable heterophil numbers in the spleen. There is published evidence that the heterophil:lymphocyte ratio varies between birds and is highly heritable (Campo and Davila, 2002). It also varies greatly amongst chicken breeds (Bilkova et al., 2017) and avian species (Minias, 2019). We did not observe any corresponding changes in circulating myeloid cells in these birds (**Figure 3**) so this phenomenon appears specific to the spleen.

Several transcripts varied substantially and idiosyncratically but did not form part of larger co-expressed clusters. Of particular interest are the Class II MHC genes *BLB1* and *BLB2*, which are polymorphic in birds and strongly-linked to disease resistance (Parker and Kaufman, 2017). Both transcripts were highly-expressed and varied more than fivefold between individuals. Despite their chromosomal proximity, their expression levels were not correlated with each other. *BLB1* did not form part of a co-expression cluster. It is normally expressed in intestine but variable expression in spleen has been reportedly associated with particular MHC haplotypes (Parker and Kaufman, 2017). *BLB2* clustered with only two other transcripts (Cluster 690): *CD1B*, one of two CD1 genes in the chicken MHC (Salomonsen et al., 2005) and LOC101747454. The latter is also described as *BLB2* in the NCBI database (https://www.ncbi.nlm.nih.gov/gene), but apparently expressed more highly than the gene annotated as *BLB2*. There are additional genes closely-related to *BLB2* around 400kb distal on chromosome 16 (Parker and Kaufman, 2017) that might produce some ambiguity in mapping. By contrast to the class II MHC transcripts, other MHC-associated transcripts (the BLB2-like *DMB2* transcript which is commonly co-expressed with *BLB2* (Parker and Kaufman, 2017), the class 1 MHC transcript *BF1* and the antigen processing transporter gene *TAP2*) varied less than 2-fold between individuals.

### Candidate Spleen Null Expression Variants

As noted above, most transcripts with extreme ranges of expression lacked informative annotation and may be expressed retroviruses or other non-coding RNA elements. There were few protein-coding transcripts that were absent or minimal in only a subset of birds. The relative absence of such variation supports the conclusion that stop-gain mutations in the founder birds are either false-positives or are not sufficiently severe to drive nonsense-mediated mRNA decay. Individual profiles of transcripts affected by candidate null expression alleles are shown in **Figure S1** and discussed below.

The gene encoding the classical Th2 T cell lymphokine, IL4, was heterogeneously expressed and the profile of variation suggested the existence of a null allele. In three birds, *IL4* mRNA was barely detected, and expression in the remaining birds fell into two groups consistent with 1x and 2x functional alleles. There is little functional data on IL4 in birds, although a recent study reported an anti-IL4 antibody and demonstrated that IL4 can drive alternative functional states in chicken macrophages (Chaudhari et al., 2018). Genetic variation in the region of the *IL4* gene has been associated with feather pecking behavior in layers (Biscarini et al., 2010)

The small MAF-related transcription factor gene, *MAFF*, also shows a spread of expression that is suggestive of Mendelian segregation of a null expression allele. Multiple members of the Maf transcription factor family are expressed during chick embryogenesis with partly over-lapping distributions (Lecoin et al., 2004). In the chicken expression atlas (Bush et al., 2018) *MAFF* is most highly expressed in macrophages. As discussed below, similarly diverse expression was detected in bone marrow-derived macrophage data.

One other example of extreme variation in splenic expression with regulatory potential is *GNAS*, encoding a stimulating subunit of G protein coupled receptors, which is a complex imprinted locus in humans. Paternally-inherited mutations in this gene in humans lead to a progressive heterotopic ossification (Bastepe, 2018). Enforced expression of a dominant negative form of GNAS in chicken somites led to rapid ectopic bone and cartilage formation (Cairns et al., 2013). Genetic variation in the *GNAS* region is associated with body weight, muscle meat quality and bone strength QTL in broilers (see Animal QTLdb). The impact of *GNAS* mutation in humans is suggestive of possible roles of GNAS in so-called wooden-breast, a pathology prevalent in high breast-yield broilers (Chen et al., 2019). One other transcript that is highly variable and might conceivably be associated with selection for a production phenotype encodes Islet2 (*ISL2*) which in mice regulates the generation and migration of specific motor neurons (Thaler et al., 2004).

Finally, phosphoserine phosphatase (PSPH) which varied >50 fold between individuals encodes a well-known mediator of L-serine biosynthesis in a variety of tissues. In laying birds, *PSPH*mRNA and protein levels were reportedly increased in the glandular and luminal epithelial cells in the developing oviduct of chicks treated with exogenous estrogen (Lee et al., 2015).

### Analysis of Variation in Gene Expression in Bone Marrow-Derived Macrophages Derived From Adult Birds of Parental Broiler and Layer Lines

The advantage of using BMDM for the current purpose is three-fold. Firstly, by contrast to spleen, they are a relatively pure cell population, which increases the sensitivity of detection and should reduce the likelihood of detecting changes in cell populations as opposed to allelic regulation of gene expression in RNAseq data. Secondly, the prolonged *in vitro* culture under defined conditions reduces the potential impact of environmental variation so that differences are more likely due to genetic variation. And finally, these cells respond to stimulation with the TLR4 agonist, lipopolysaccharide (LPS) with a profound change in gene expression. We can therefore monitor the impact of genotype on inducible genes. In a previous study, we were able to identify the differential expression of Z chromosome-associated transcripts in male versus female BMDM, and we inferred that macrophages from female birds have a novel mechanism to compensate for the presence of the inducible interferon genes on the Z chromosome (Garcia-Morales et al., 2015).

The previous study (Garcia-Morales et al., 2015) used gene expression microarrays to quantify mRNA levels and was limited by the available annotation at the time. We first sought to repeat the earlier study and to compare males and females and commercial layers and broilers. The analysis of outbred commercial birds from the parental lines provides a control for the greater expression diversity that we anticipate in F2 birds from deliberate inbreeding. BMDM from three adult female and three adult male broilers and three adult layer females were cultivated with or without LPS for 24h. The primary data are provided in **Table S3**. The prolonged incubation was chosen to avoid temporal differences in the rate of response and specifically to focus on late-response genes that require stimulation by autocrine interferon signaling. One disadvantage is that the dataset does not capture the acute pro-inflammatory and anti-inflammatory transcripts that are induced transiently by LPS. This transiently-induced set includes the negative feedback regulator IL10. We showed previously that autocrine IL10 inhibits LPS-inducible cytokine production in BMDM (Wu et al., 2016). *IL10* was only detected at low levels in the 24 hour-stimulated BMDM (<10 TPM) where the receptor gene, *IL10RA*, was highly-expressed and further induced by LPS.

The expression of known macrophage-specific genes (Bush et al., 2018) including *CSF1R* and the transcription factor *SPI1* was high and invariant among all the samples, supporting the consistency and relative purity of these macrophage populations. Amongst the averaged data for the 8461 transcripts that were expressed >10 TPM in at least 1 BMDM sample, 872 were induced >2-fold and 697 were repressed >2-fold by LPS. The most highly-inducible genes included LPS-responsive transcription factor genes *BATF3*, *HIF1A*, *IRF1*, *IRF8* and feedback regulators including *CISH*, *SOCS3* and *TNFAIP3*. Chicken BMDM, like BMDM from rodents, take up arginine and produce large amounts of nitric oxide in response to LPS (Wu et al., 2016). Accordingly, *NOS2* and *GCH1* (which is required to generate the NOS2 co-factor tetrahydrobiopterin) and the citrulline-arginine recycling enzymes, *ASL2* and *ASS1*, were each induced by LPS in all cultures. Unlike rodent BMDM, which induce the cationic arginine transporter *SLC7A2* in response to LPS (Young et al., 2018) chicken BMDM induced a distinct arginine transporter, *SLC7A3*. One other important difference between chicken and rodent BMDM is the expression of *IRG1*, now annotated as aconitate decarboxylase (*ACOD1*). In mammalian macrophages, *ACOD1* was profoundly-induced by LPS. Inducible *ACOD1* has been attributed roles in metabolic reprogramming in stimulated macrophages, subverting the TCA cycle by diverting iso-citrate and catalyzing the generation of cis-itaconate, which has a proposed anti-inflammatory feedback function (Mills et al., 2018). In the chicken BMDM, *ACOD1* was already highly-expressed in the unstimulated state albeit induced further by LPS. Interestingly, *ACOD1* polymorphism has been linked to resistance to the macrophage-tropic pathogen Marek’s disease virus (Smith et al., 2011). One transcript of particular interest is *IGF1*, encoding a major regulator of somatic growth which is associated with a signature of selection in broilers (Qanbari et al., 2019). In mammals, *IGF1* is highly-expressed in macrophages and regulated by CSF1 (Gow et al., 2010) but the expression in chicken BMDM was below the detection limit.

The LPS-repressed genes include cell cycle-associated transcripts, such as *BUB1*, *FOXM1* and *MKI67* (which encodes the commonly-used proliferation marker KI67), reflecting the known ability of LPS to inhibit cell proliferation in this culture system. Associated with this growth inhibition, as in mammalian macrophages (Yue et al., 1993), LPS stimulation down-regulated expression of *CSF1R*. LPS treatment repressed other transcripts encoding multiple cell surface receptors and secreted effectors to a much greater extent than *CSF1R*. High-expression transcripts reduced >10-fold included membrane receptors *TREM2* (and related *TREMB1* and *TREMB2*), *GPR34, ITGB5, MARCO, TLR2A, TLR2B, TLR7*, and *ENSGALG00000028304* (encoding the macrophage mannose receptor/KULO1 antigen (Hu et al., 2019).

Differences in regulation between males and females are considered further below, and in these commercial birds, we sampled only three male broilers. The expression of transcripts on the Z chromosome is shown on a separate sheet in **Table S3**. It is nevertheless striking that of 380 Z chromosome-encoded transcripts detected in BMDM with expression levels of >10 TPM, <10% were up or down-regulated by LPS.

**Figure 4:**
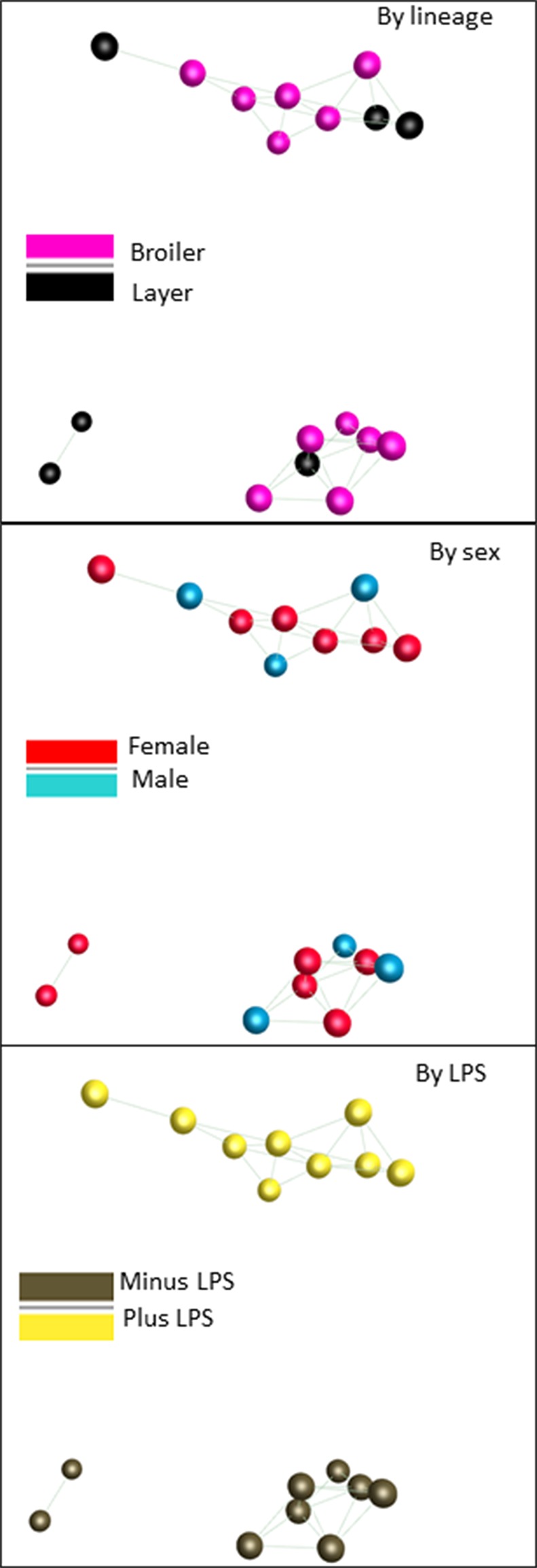
Network analysis of the response of BMDM generated from adult broiler and layer chickens to LPS. RNAseq gene expression data from BMDM cultured with or without LPS for 24h was analyzed using the network visualization tool Graphia. The panels show the sample-to-sample matrix with a Pearson correlation threshold of 0.97. In the three panels birds are identified based upon breed, sex or treatment as indicated. Note that the samples segregate solely based upon LPS stimulation. The sets of LPS-regulated transcripts that contribute to this separation are summarized in **Table S3**.

**Table S3** also shows the range of expression for the control and LPS-stimulated samples. There was considerably greater variation than observed in the spleen RNAseq data. 615 transcripts varied across >5-fold range in the unstimulated data and 1110 by >5-fold across the LPS-stimulated samples. To explore this variation further and seek evidence of co-regulated genes we again used Graphia. **Figure 4** shows a sample-to-sample network graph (r ≥ 0.97) for these data. This shows that there was no segregation of the broilers from layers, suggesting that there is no consistent strain-specific gene expression pattern. Consistent with the evidence of profound LPS-induced changes in gene expression, the main axis of separation is driven by LPS stimulation which was analyzed in more detail in the F2 progeny.

**Table S3** also summarizes the fold changes comparing the broilers and layers in the control and LPS-stimulated states. Consistent with the network analysis, there are few annotated transcripts, and even fewer highly-expressed transcripts, that distinguish the expression profiles based upon breed. The most broiler-enriched transcripts of interest are *S100A8*, *CCL5* and *ETS2*, whilst the layers had higher expression of *CMPK2, SLC40A1, APOA1, IFIT5, STAT1* and *MMP9*.

### Analysis of Gene Expression in BMDM Generated From Progeny of F1 Brother-Sister Matings

To continue to address the hypothesis that sibling-mating would expose homozygosity for high and low expression alleles and amplify the variation seen in the commercial broiler and layer lines, we isolated bone marrow from a total of 32 hatchlings from different families, grew BMDM, treated them with or without LPS as above for 24h, isolated mRNA and profiled gene expression by RNAseq. As with spleen, we chose hatchlings to avoid possible confounding influences of pathogen exposure including routine immunization with live vaccines. We also hoped to validate a method that would enable early and rapid screening of the progeny of defined matings that might form the basis of breeding decisions. A sample-to-sample analysis of the complete dataset was performed using Graphia and four samples from stimulated and unstimulated states were identified as major outliers and were removed. To enable pairwise-comparisons of stimulated and unstimulated states, we further removed those samples for which there was not a pair, leaving a total of 28 F2 birds from 6 separate families for analysis (+/- LPS). This is a proof-of-concept experiment and it was beyond our resources to survey the full genetic diversity within the founders. To maximize the likelihood of detecting multiple F2 birds with the same expression variant inherited from a grandparent mating we included 16 birds from 4 F1 brother-sister matings from the same grandparent cross. They are in effect double-cousins. We also included six birds from family H (which exhibited poor hatch rate, low weight at hatch and poor survival (**Figure 2**) to explore possible detection of deleterious variants in this sibship, and a smaller number of birds from other grandparental matings to include broiler-layer specific variant *CSF1R* alleles.

Initial analysis of the expression data revealed variable detection of multiple genes associated with mesenchymal lineages including numerous collagen genes. These transcripts were not detected in the BMDM from adult commercial birds in **Table S3**. Neonatal calvarial cultures are routinely used to generate osteoblasts in mice. Such cultures contain large numbers of macrophages even without addition of growth factors (Chang et al., 2008). The FANTOM consortium recently published an analysis of transcriptional regulation of chicken promoter during development, including a sample annotated as bone marrow-derived mesenchymal stem cells which is actually a hatchling calvarial bone marrow culture (Lizio et al., 2017). This sample exhibited abundant expression of known macrophage-specific genes including *CSF1R* alongside multiple collagens. Accordingly, we concluded that the hatchling bone marrow (unlike adult) probably contains mesenchymal stem cells which proliferated and differentiated alongside the macrophages in our culture system. In mouse calvarial cultures the macrophages and osteoblasts interact with each other to control calcification, an interaction that is paralleled *in vivo* (Chang et al., 2008). The culture system therefore unexpectedly enabled us to examine possible mesenchyme-associated gene expression variants that are quite likely relevant to the phenotypic diversity in the broiler-layer cross, but such an analysis required deconvolution of the data.

We first considered the sets of control and LPS-stimulated samples separately (**Table S4**) and calculated the ratio of maximum/minimum expression. The extent of variation amongst protein-coding transcripts was massively greater than in the spleen or adult broiler and layer BMDM data in **Table S3**. To identify possible null (absolute loss of expression) variation, we identified the set of transcripts for which the maximum was >20 and minimum <1. **Table S4** includes a Venn diagram for the control and LPS-stimulated states. Of a total of 962 transcripts that met the criterion for extreme variation between individuals, 365 (39%) overlapped between the two sets (control, + LPS) and 432 (45%) were specific for the LPS-stimulated state. The set of variable expression transcripts that is independent of LPS stimulation is clearly enriched for mesenchyme-associated genes including 10 separate collagen genes. Some of these transcripts (e.g. *COL1A1*, *COL1A2*) appear in the LPS-stimulated list only because they are marginally above the detection limit (>1 TPM) in the lowest-expressing sample. Nevertheless, the analysis validates some of the conclusions from the spleen data suggesting the existence of effective null expression alleles for GNAS, *IL4* and *MAFF*. Furthermore, *CSF3*, which was profoundly LPS-inducible in the large majority of birds, was barely detected in others. Such variation could contribute to extreme variation in heterophils in the spleen (**Table S2**).

The expression of *CSF1R* mRNA in the F2 hatchling birds was around 30% lower than in the BMDM cultures from adult commercial birds (221 versus 321 average TPM), but down-regulated to a similar value (145 versus 132 average TPM) in the LPS stimulated cultures. The level of *CSF1R* mRNA varied over a much greater range (47-347 TPM) in the F2 BMDM cultures compared to the commercial birds.

**Figure 5:**
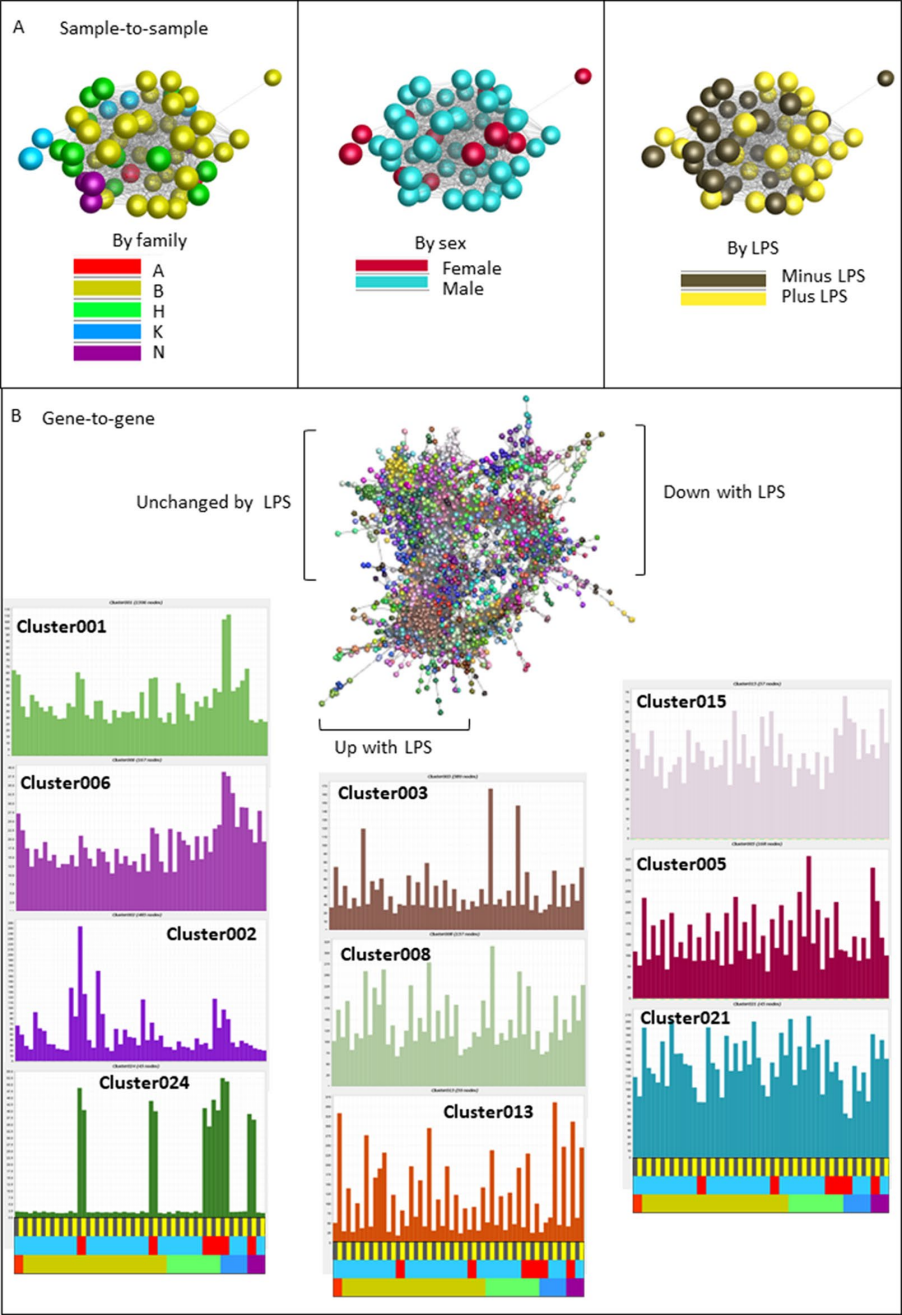
Network analysis of the response of BMDM generated from F2 inbred hatchling chickens to LPS. RNAseq gene expression data from BMDM cultured with or without LPS for 24h was analyzed using the network visualization tool Graphia. (Panel **A**) shows the sample-to-sample matrix at a Pearson correlation threshold of 0.97. Note that there is no clear separation based upon family or sex, and less segregation based upon LPS treatment than in the parental comparison (**Figure 4**). (Panel **B**) shows the gene-to-gene matrix generated at Pearson r ≥ 0.85 with clusters of co-regulated transcripts colored. This analysis reveals a clear segregation of clusters that are increased, decreased or unchanged by LPS. The average profiles of selected clusters discussed in the text are shown in the surrounding histograms. The color code at the bottom of each column indicates LPS versus control in pairs from the same birds, sex, or family (colors as in Panel **A**). The transcripts contained within each Cluster are shown in **Table S4** and **Table S5**.

We next deconvoluted the data by network analysis using Graphia. We anticipated that transcripts associated specifically with gene expression in the separate mesenchyme and macrophage populations would form separate clusters and their relative levels would provide a surrogate for the relative purity of each cell culture. Network graphs for this dataset are shown in **Figure 5**. The sample-to-sample profile (**Figure 5A**) is color-coded for family (left), sex (middle) or treatment (right). The samples did not separate based upon sex and there was also no obvious segregation based upon family or the parent allelic variants selected for analysis. By contrast to the BMDM data from the commercial birds there was also no separation based on LPS stimulation. The average profiles of the largest clusters derived from the gene-to-gene analysis (r ≥ 0.85) are shown in **Figure 5B**. Key genes and functional annotation terms for the larger clusters are summarized in **Table 3** and the full gene lists in each cluster are provided in **Table S5**.

**Table 3:** Functional annotation of clusters derived from untreated and LPS treated F2 BMDM.

Cluster Number (number of nodes)	Expression pattern	Representative genes	Enhanced terms (P value)
001 (1396)	Unchanged by LPS	*ACTA2, ADAMTSs, ASPH, COL1A1, COL1A2, FBN1, FN1, MFAPs, TGFB3, TGFBR2/3, THBS1, THBS2*	Protein processing in endoplasmic reticulum (5.5E-10); Poly(A) RNA binding (3.2E-8); Extracellular matrix (1.6E-6); Endoplasmic reticulum (8.7E-6); ER-Golgi transport (7.5E-5); Focal adhesion (1.5E-4)
002 (485)	Unchanged by LPS	*COL9A1, COL10A1, COL11A1, FGFRs, FOXO6, IRX5/6, MEF2A/B/C, PAX3, RUNX2, SIX2/3, SMAD1, SOX5/8/9*	Extracellular matrix (0.003); Secreted (0.007); Glycoprotein (0.012); Signal (0.038)
003 (389)	Up with LPS	*ATF3, BHLHE40,* C*D274, CEBPB, FOSL2, GGCL1, GVIN1, IFIT5, IFIH1, IRF1, IRF8, IRF9, OASL, SOCS1*	Zinc finger, RING/FYVE/PHD-type (0.02); Influenza A (0.01)
004 (259)	Unchanged with LPS	*CPSF2, DDX6, LEO1, PAPD5, PAPOLA, PARN, PRPF38B, PRPF39, PRPF40A, PRPF6, SMU1, TOP2B, TOP3B, TOPBP1*	Spliceosome (2.4E-9); Cytoplasmic mRNA processing body (0.0004)
005 (168)	Down with LPS	*C3AR1, CD14, FAAH, MAOA, PEX16, PSEN2, TNFAIP8L1, TUBB1*	Transmembrane helix (0.1); Aldolase-type TIM barrel (0.07)
006 (167)	Unchanged by LPS	*BUB1, BUB1B, BUB3*, centromere protein genes, kinesin genes, kinetochore complex components	Cell cycle (4.9E-14); Mitosis (9.8E-13); Cell division (2.3E-11); Centromere (8.3E-11)
007 (160)	Unchanged by LPS	*RPL* and *RPS* genes, translation initiation and elongation genes	Structural constituent of ribosome (3.6E-81); Ribosome (4.7E-79); Translation (1.2E-68); Protein biosynthesis (1.8E-7)
008 (137)	Up with LPS	*BACH1, IRF6, KLF8, MAFK, REL, TLR15*	No significant enrichment
009 (100)	Down with LPS	*IGF2, KLF3, KLF13, MAF1, TAF12*	No significant enhancement
010 (76)	Unchanged by LPS	*AAR2, CNOT10, EXOC1, GABPA, POLR1A*	RNA recognition motif (0.002)
011 (71)	Unchanged by LPS but one animal very high	*ACTG2, CDH11, COL7A1, ENPP2/3, FGF7, NPY, PDGFRA, VAV2*	Calcium ion binding (0.2)
012 (65)	No trend with LPS	All unannotated *ENSGALG*	
013 (59)	Up with LPS	*CCL4, BATF6, FLT1, IL13RA1, IL1B, NFKB1, NFKB2, NFKBIA, TRAF2, TRAF3, ZC3H12A*	Toll-like receptor signaling pathway (0.0005); Cytosolic DNA sensing pathway (0.001); RIG-I-like receptor signaling pathway (0.001); Inflammatory response (0.01)
014 (57)	Up with LPS	*CCNE1, FMR1, SMCHD1, TRAF6, USP16*	Cell cycle (0.1)
015 (57)	Down with LPS	Mostly unannotated *ENSGALG* and *LOC* genes	No significant enhancement
016 (52)	Down with LPS	*ATP6AP1, ATP6AP2, ATP6V0D1, CASP9*	No significant enrichment
017 (52)	Unchanged with LPS	*ACTR1A, COG5, E4F1, EIF2AK4, INSIG2*	No significant enrichment
018 (52)	Variable response to LPS	All unannotated *ENSGALG*	
019 (51)	Variable response to LPS	*ACOD1* (*IRG1*), *IRF2, MAP2K3, TAOK1, TBK1, USP15*	Protein ubiquitination involved in ubiquitin-dependent protein catabolic process (0.01)
020 (45)	Unchanged with LPS	*CCT* genes, *HSP* genes	Positive regulation of protein localization to Cajal body (1.5E-10); Chaperonin TCP-1, conserved site (4.7E-10); Positive regulation of establishment of protein localization to telomere (2.7E-10); Chaperone (2.1E-9)
021 (45)	Down with LPS in most	*CSF1R, IL2RG, IL6R, TLR4*	No significant enrichment

Benjamini Hochberg corrected P values are presented. First 21 clusters only as number of genes becomes too low for meaningful analysis in smaller clusters. This analysis used DAVID (https://david.ncifcrf.gov/home.jsp) to determine enrichment for annotation terms.

### Mesenchyme-Related Gene Expression in F2 Bone Marrow Cultures

Cluster 1 which contains 1396 genes, includes major bone-associated collagens (*COL1A1*, *COL1A2*) and extracellular matrix proteins alongside multiple cell cycle-associated transcripts, and is enriched for GO terms associated with mesenchyme and extracellular matrix. This cluster of genes most likely reflects the presence of varying numbers of proliferating mesenchymal cells. There are around 180 transcripts with no current informative annotation which can be inferred to be related to mesenchyme differentiation. The expression of mesenchyme-associated transcripts was not regulated by addition of LPS in any of the birds. Given that one grandparent was a broiler, we suggest that differential growth of mesenchymal cells in culture is related to selection for growth and muscle/bone/fat related production traits. If there is a genetic basis for the variable growth of mesenchymal cells in this culture system, it is likely to involve regulated expression of growth factors or growth factor responsiveness. Transcripts encoding members of each of the many families of growth factors implicated in mesenchymal stem cell (MSC) growth and differentiation (e.g. *BMP4, BMP6, CTGF, FGF13, INHBA, NOTCH2, PDGFB, TGFB3, VEGFA*, and *WNT5B*) were each highly-expressed and varied greatly between individual birds (**Table S5**). Most were not contained within cluster 1, but the cluster does contain transcripts encoding multiple growth factor receptors (e.g. *ACVR1, ACVR2A, DDR2, EPHA3, EGFR, PDGFRB, SMO, TGFBR2*) and an equally plausible mechanism is regulated expression of these receptors. Cluster 1 also contains *TGFB3* and *THBS1* (thrombospondin 1) both of which control MSC proliferation in humans (Belotti et al., 2016). Genes within this cluster are candidates for causal association with broiler production traits. Consistent with that view, *ASPH* lies within a QTL interval associated with muscle development on chromosome 2 (Godoy et al., 2015) and *THBS2* has been identified as a candidate gene within a QTL for fatness in chicken (Moreira et al., 2015).

Cluster 2 is a distinct mesenchyme cluster that varies to a much greater extent between birds than cluster 1, and in the majority of birds the average expression of transcripts within the cluster was down-regulated by LPS. The GO enrichment indicates an association with extracellular matrix and secreted proteins, including collagens associated specifically with hypertrophic chondrocytes (e.g. *COL9A1*, *COL10A1* and *COL11A1*), four FGF receptor family members, and many other transcripts associated with hypertrophic chondrocytes in mammals. Cluster 2 also contains many known transcriptional regulators of chondrocyte development in mammals [reviewed in (Liu et al., 2017)] including *IRX5* and *IRX6*, *MEF2A*, *B* and *C*, *PAX3*, *RUNX2*, *SIX2* and *SIX3*, *SMAD1*, *SOX5*, *SOX8* and *SOX9*. The coordinated regulation of these factors suggests that the basic biology of chondrocyte differentiation is conserved in birds, but also that the individual birds/culture differ greatly in their support of this pathway. There is at least one obvious candidate regulator in this cluster. Chondromodulin (*CNMD*) is required for the maturation of chondrocytes and expression of *Col10a1* in mice (Yukata et al., 2008) and was amongst the most divergent transcripts in the F2 cultures (**Table S5**). We suggest that genes within clusters 1 and 2 are likely candidates underlying growth and composition traits in broilers.

### Variable Expression of LPS-Regulated Genes in BMDM From F2 Inbred Birds

LPS signals to macrophages through the receptor TLR4. The response of macrophages to LPS involves two distinct adaptor proteins, MYD88/TIRAP and TRIF/TRAM and downstream target genes can be classified based upon their dependence on these two effector pathways. The TRIF/TRAM pathway links to induction of type 1 interferon (IFN) and an autocrine stimulatory cascade. Until recently it was claimed that LPS-stimulated chicken macrophages do not produce endogenous IFN. However, a recent study (Ahmed-Hassan et al., 2018) reported the release of type 1 IFN activity from LPS-stimulated macrophages and provided evidence of autocrine signaling. They were not able to detect the induction of *IFNB* mRNA. *TLR4* is highly polymorphic at the protein-coding level in chicken (Swiderska et al., 2018). Our F2 dataset contains one bird (B628) in which *TLR4* mRNA was exceptionally low in both control and LPS-stimulated states. *TLR4* is part of a small cluster (cluster 21) that also includes *CSF1R* but does not include any other macrophage-specific genes. Neither *CD14* (encoding the TLR4 co-receptor), nor *MYD88* or *TIRAP* varied substantially between birds. Accordingly, we conclude that this bird was specifically deficient in *TLR4* expression.

The candidate targets of the TRIF/TRAM pathway are regulated by transcription factors of the IRF family. Cluster 3 contains the largest set of transcripts that was up-regulated by LPS. It is much smaller than the equivalent in commercial birds because of tighter correlations generated by the larger dataset, and because the level of induction varied substantially between individuals. The likely driver of the variation between birds is differential regulation of the LPS-inducible transcription factors, *IRF1*, *IRF8* and *IRF9* as well as *ATF3*, *BHLHE40*, *CEBPB* and *FOSL2*. In human monocytes, eQTL analysis of the LPS-inducible gene expression response revealed *trans*-acting variants impacting upon the regulation of the suite of genes regulated by inducible autocrine IFN signals (Fairfax et al., 2014). *IRF1* and *IRF8* are obligatory intermediates in induction of many LPS-inducible genes in mouse macrophages ((Roy et al., 2015) and references therein) and in chickens *IRF1* is a key mediator of type 1 IFN antiviral signaling (Liu et al., 2018). Cluster 3 contains many known IFN-inducible effector genes amongst which the most highly-expressed/inducible and hypervariable include C*D274* (encoding the check point inhibitor PD-L1*), GVIN1, GGCL1, IFIT5, IFIH1, OASL* and the feedback inhibitor *SOCS1*. We can infer that the other transcripts in this cluster, including those that are poorly annotated, form part of the IFN effector system. *IRF1* mRNA was induced in all but one of the birds, but the stimulated level of expression varied 250-fold amongst birds. The induction of *IRF1* in mouse and human macrophages is triggered by transient expression and autocrine signaling by IFNB1, acting through the type 1 interferon receptors, IFNAR1 and IFNAR2 (Sheikh et al., 2014). Both IFN receptor genes were robustly expressed in all the BMDM preparations with relatively little variation. However, birds lack *IRF3*, the key upstream regulator of *IFNB1* induction in mammals, and some evidence places *IRF1* upstream of *IFNB1* induction in chickens (Liu et al., 2018).

There are several other clusters containing IFN-associated genes that are distinct from cluster 3. Cluster 8 contains *IRF6* alongside other inducible candidate transcriptional regulators, *BACH1, KLF8, MAFK* and *REL*. One notable gene within this cluster is *TLR15*, a member of the TLR1 family that is unique to birds and reptiles and implicated in response to fungal/yeast pathogens (Boyd et al., 2012). Most transcripts in cluster 19 were expressed constitutively and induced further by LPS. This cluster includes *IRF2*, transcripts encoding signaling molecules (*TAOK1, TBK1, MAP2K3*) and the feedback inhibitor *USP15*. Cluster 19 contains the profoundly-inducible *IRG1* gene (now annotated as *ACOD1*) discussed above. The transcriptional regulator *IRF7* is in a much smaller cluster, cluster 35, along with the genes for both IL23A and IL12B, which form the functional dimer of the proinflammatory cytokine IL23, and the chemokine CCL20.

The IFN genes in chickens are on the Z chromosome and remain poorly annotated (Goossens et al., 2013) and in any case, the pulse of endogenous *IFNB1* that occurs in stimulated mammalian macrophages is transient (Liu et al., 2018) and would not have been captured in this time course. Our data highlight that autocrine IFN induction occurred in the response of chicken BMDM to LPS, but we cannot determine definitively whether the >100-fold variation in IFN target gene expression we observed is in part due to variation in IFN induction. One hint can be gained from the fact that the F2 birds are a mixture of males and females. The individual male and female samples are highlighted in **Table S4** and the male/female ratio in gene expression is calculated. *IRF1* and many of the known target genes were each expressed significantly more highly in the LPS-stimulated cultures of the male birds, suggesting that there is a correlation between their expression and variation in IFN production. In mice *IRF1* and *IRF8* are regulated independently and cooperate in induction of subsets of interferon target genes in macrophages (Langlais et al., 2016). The cluster analysis indicated that *IRF2*, *IRF6*, and *IRF7* vary independently of *IRF1*, each controlling a subset of IFN target genes. We suggest that there are regulatory variants strongly impacting the IFN pathway at multiple levels. As shown in **Figure 1C**, the broiler and layer founder birds can be distinguished based upon SNPs associated with the IRF loci alone, suggesting that selective breeding has also selected variation at each of these key regulators. **Table S6** lists all of the variants detected in the founder birds at each of the IRF loci. They include 15 SNPs within the 1kb promoter region of *IRF1* and 24 within the 1kb promoter region of *IRF7*. There are also several non-synonymous coding variants in *IRF7* that are enriched in the broilers but are not predicted to be deleterious.

MYD88/TIRAP1 activation is connected through IRAK1/4, TRAF6 and the kinase TAK1 to activation of the transcription factor NFKB. Most NFKB target genes in macrophages are induced transiently and subsequently repressed by the combined actions of numerous feedback repressors (Baillie et al., 2017). Cluster 13 is the largest cluster that contains LPS-inducible transcripts likely induced by the MYD88/TIRAP-dependent pathway and includes the pro-inflammatory cytokine gene *IL1B* and the chemokine gene *CCL4*. This cluster contains transcripts encoding a number of known feedback regulators of the response including *BATF3*, *NFKIA*, and *ZC3H12A*. As evident from the averaged profile, the level of induced expression of this cluster of genes is much less variable between individuals. It is also not significantly different between the males and females. Hence, there is clearly separate regulation of the two gene sets lying downstream of MYD88/TIRAP and TRIF/TRAM.

The reciprocal to the LPS-inducible gene sets is the clusters of genes that are repressed by LPS. These clusters are not highly variable between individuals. The GO term enrichments are summarized in **Table 3**. Cluster 6 is very significantly enriched for known cell cycle-related transcripts and cluster 7 for components of the ribosome and translation apparatus, both likely reflecting inhibitory effects of LPS on cell proliferation in macrophages, and possibly also in the mesenchyme component of the culture.

Although the macrophage content of the cultures likely varies inversely with the mesenchymal content, we do not detect a macrophage-specific cluster. *CSF1R* forms part of small cluster (cluster 21) alongside *TLR4* that is, as expected, also down-regulated by LPS, whilst the core macrophage-specific transcription factor gene, *SPI1* does not correlate with any target genes (Cluster 80). We interpret this to indicate that there is considerable cis-acting variation in the large majority of macrophage-specific genes so that correlations with other macrophage-expressed transcripts fall below the threshold chosen.

### Analysis of the Low Fitness F2 Family (Family H)

As mentioned above, one family showed consistently low weight at hatch (**Figure 2**). This family also had a low hatch rate and three individuals died at or just before hatch with abnormalities in the brain, cartilage and muscle seen at autopsy. We compared gene expression in BMDM from this family to that in all other F2 birds, before and after exposure to LPS, to determine whether this family showed altered expression patterns. In the untreated samples, DAVID analysis showed that genes that were lower in Family H included those with GO terms relating to signal transduction, skeletal system development, collagen, extracellular matrix, chondrocyte differentiation. GATHER analysis confirmed the association with skeletal and cartilage development and cell signaling. Since expression of connective tissue genes reflects in part contamination of BMDM with mesenchymal cells (as discussed in detail above), these results may suggest a deficiency in connective tissue formation or the interaction of these cells with macrophages. For genes that were higher in Family H, DAVID found slight enrichment for GO terms associated with receptors, while GATHER detected GO terms associated with response to stimulus, defense response and immune cell activation.

After LPS stimulation of BMDM, there was enrichment for immunoglobulin and extracellular matrix GO terms and GATHER found an association with cell signaling, metabolism and ion transport in the genes that were lower in Family H than the other samples. Among genes that were higher in Family H after LPS stimulation there was enrichment for terms related to the response to LPS and apoptosis (DAVID analysis). GATHER also found association with terms for cell death/apoptosis as well as terms relating to bone formation. The results are presented in **Table S7**.

These results suggest that the poor hatch rate, low hatch weight and early mortality in Family H was related to a failure of normal development due to low expression of key morphological genes which may indicate a deficiency of mesenchymal cells in the bone marrow. There may have been concomitant infection with perturbed expression of genes of the immune system. The Family H BMDMs may have had a greater response to LPS since GO terms associated with the response were higher in these cells.

### Candidate Null Expression Alleles

The set of candidate genes showing extremes of expression (including candidate nulls) in **Table S4** clearly reflects in part the variable contribution of mesenchyme lineage cells to the cultures. However, there are a number of genes that were highly-expressed, or selectively-induced in an entirely gene-specific manner. Selected examples are shown in **Figure S2**, which illustrates that each has a different pattern of variation between individuals. *ENSGAL00000028304* was recently shown to encode a macrophage mannose receptor (MRC1L-B, now annotated as MMR1L4), recognized by antibody KUL01 (Staines et al., 2014). KUL01 is a widely-used monocyte-macrophage marker. There are multiple members of this family encoded by the chicken genome, but *MRC1L4* is the only one expressed in BMDM. It forms part of a small cluster (cluster 48) that also includes *TLR2A*. Pentraxin 3 (PTX3) in mammals is required for effective host defense against influenza infection (Reading et al., 2008). In chickens PTX3 is as an acute phase marker of bacterial infection that was undetectable in spleen (consistent with our data) but induced rapidly by infection (Burkhardt et al., 2019). *PTX3* was highly-expressed in control BMDM and regulated only marginally by LPS. The expression varied over 3 orders of magnitude between individuals and was not correlated with any other gene.

Previous studies of induction of pro-inflammatory cytokines in heterophils of birds selected for resistance to *Salmonella* revealed a positive correlation between resistance and induction of *IL6* and *IL8* (Swaggerty et al., 2004). The induced levels of these two genes also varied greatly between birds in our study and were not correlated with each other. There have, to our knowledge, been no published studies of *CSF3* regulation in chickens. *CSF3* was massively induced by LPS and clustered with putative IRF1/8/9 target genes in cluster 3. The extent of variation, with several birds showing almost undetectable expression, indicates that there are either *cis*-acting variants or that *CSF3* is regulated by multiple transcription factors within cluster 3. Regardless of mechanism, *CSF3* variation likely underlies variation in the heterophil response to infection.

We noted in the discussion of splenic gene expression that the class II MHC gene, *BLB2* showed evidence of variation between individuals. Chicken BMDM are strongly Class II MHC-positive but *BLB2* expression varied between birds. *BLB2* expression varied in parallel with *CD74*, which encodes the class II-associated invariant chain. Since these are on different chromosomes, there is likely to be an upstream *trans*-acting regulator. The obvious candidate, *CIITA*, was not detected in our annotation. Two other genes of interest were *IL4* and *IL34*. In keeping with the findings from the spleen discussed above, *IL4* was detected in a subset of preparations, unaffected by LPS, and undetectable in others. *IL34* was barely detectable in BMDM. *CSF1*, encoding the macrophage growth factor, was expressed constitutively and induced by LPS, but was also very variable amongst samples.

## Conclusion

The main purpose of this study was to demonstrate that allelic imbalance in gene expression and/or coding variants of large effect in an outbred commercial chicken population could be uncovered by brother-sister mating of F1 progeny to generate an array of F2 individuals, and to survey the impact of homozygosity for the possible set of variants. It was not our intention to document such variants extensively. The founder birds were chosen deliberately to be as outbred as possible and as different from each other as possible.

Despite the high prevalence of candidate null mutations in the grandparental broilers and layers, we found little evidence of adverse impacts of sibling mating on growth and development. It is likely that the highly-selective breeding of commercial birds has largely purged variants that impact on hatch-rate and fertility. Extensive inbreeding in pedigree dogs is not strongly-associated with health-related traits (Jansson and Laikre, 2014). Similarly, a large-scale survey of Irish cattle genotypes revealed relatively few examples of severely deleterious recessive alleles affecting survival or production traits (Jenko et al., 2019). A growing literature in humans, based upon analysis of populations where consanguineous marriage is common, shows that the large majority of the thousands of homozygous null mutations detected in such populations have no overt phenotype (Erzurumluoglu et al., 2016). However, if the gene function is known, more subtle phenotypes can be detected (Saleheen et al., 2017).

Like the direct measurement of gene products and their functions, analysis of mRNA levels provides an intermediate phenotype to assess the impact of homozygosity. The variation we observed in the hatchling spleen supported the hypothesis that there are allelic variants in parental broiler and layer lines that strongly impact on gene expression in immune cells. This also indicated a major difference in heterophil accumulation in the spleen evident from the correlated expression of known marker genes. In the analysis of BMDM, by comparison to the set of parental outbred commercial broiler and layer lines, the F2 birds exhibited considerably greater variation in both basal and LPS-inducible gene expression. In particular, we highlighted a set of interferon-inducible genes that was co-regulated and varied in expression across a 100-1000-fold range. We infer that this variation is associated with the extensive polymorphism in IRF family members (**Figure 1B**) that distinguishes broilers and layers. The approach we have demonstrated has some potential for use in selective breeding since it does not require mature birds or disease challenge. It is entirely plausible that the generation of homozygosity for low expression alleles that we have identified contributes to the phenomenon of inbreeding depression (Charlesworth and Willis, 2009).

If there is, as we suspect, genetic variation that impacts on IFN production encoded by the Z chromosome, the analysis of BMDM from female hatchlings could be applied as progeny testing to select high and low responder lines, assaying only for the LPS-inducible target genes in Cluster 3 from the analysis of BMDM. The culture system could also be used to assess candidate genes and the impact of putative *cis*-acting variation. For example, in the Sal1 locus affecting *Salmonella* resistance in birds, two candidate genes have been identified, *AKT1* and *SIVA1* (Psifidi et al., 2018). *SIVA1* was not detectably expressed in BMDM. Several other genomic regions underlying QTL for *Salmonella* resistance in this and previous studies [e.g. (Thanh-Son et al., 2012)], contain genes that were hypervariable amongst F2 progeny (e.g. *IRF1* and *IRF6*) in the dataset analyzed here. In principle, using the founder DNA and mRNA sequences we could infer the existence of allelic homozygosity for each of the transcripts detected in the spleen and BMDM RNAseq datasets but the size of the population analyzed here was not sufficient to separate *cis*-acting from *trans*-acting variation or to attribute causation. It remains to be seen whether the variation in bone marrow-derived mesenchyme proliferation that we uncovered *inter alia* might also provide an opportunity to accelerate phenotypic selection for production traits.

## Data Availability Statement

The variant call files (VCF) for the genomic DNA sequence are available at Edinburgh Datashare (datashare.is.ed.ac.uk) under the Data title: “Variant discovery from whole genome sequence data from a commercial broiler line and a CSFIR-mApple reporter transgenic layer line.” The primary RNAseq sequence data have been deposited in the European Nucleotide Archive under Bioprojects PRJEB22373 and PRJEB34093. The primary and processed DNA and RNA sequence data will also be made available by the authors, without constraint on use, to any qualified researcher upon request.

## Ethics Statement

The animal study was reviewed and approved by Protocols and Ethics Committees of the Roslin Institute and the University of Edinburgh.

## Author Contributions

LF, AM, RH and JO’D performed experiments and primary data analysis. SB and AG performed bioinformatic analysis of RNA and DNA sequence, respectively. AP and KB performed genetic analysis. KS performed network analysis. DH and KS analyzed the data and wrote the manuscript, with editing input from SB, AG, AP, and KB. DH conceived and funded the project.

## Conflict of Interest

The authors declare that the research was conducted in the absence of any commercial or financial relationships that could be construed as a potential conflict of interest.
