# Supplementary material for: Analysis of the Progeny of Sibling Matings Reveals Regulatory Variation Impacting the Transcriptome of Immune Cells in Commercial Chickens
Source: Front Genet. 2019 Nov 14;10:1032. doi: 10.3389/fgene.2019.01032 (PMC6870463; doi:10.3389/fgene.2019.01032)
Supplement: Supplementary file 1 [file Presentation_1.pptx]

## Slide 1
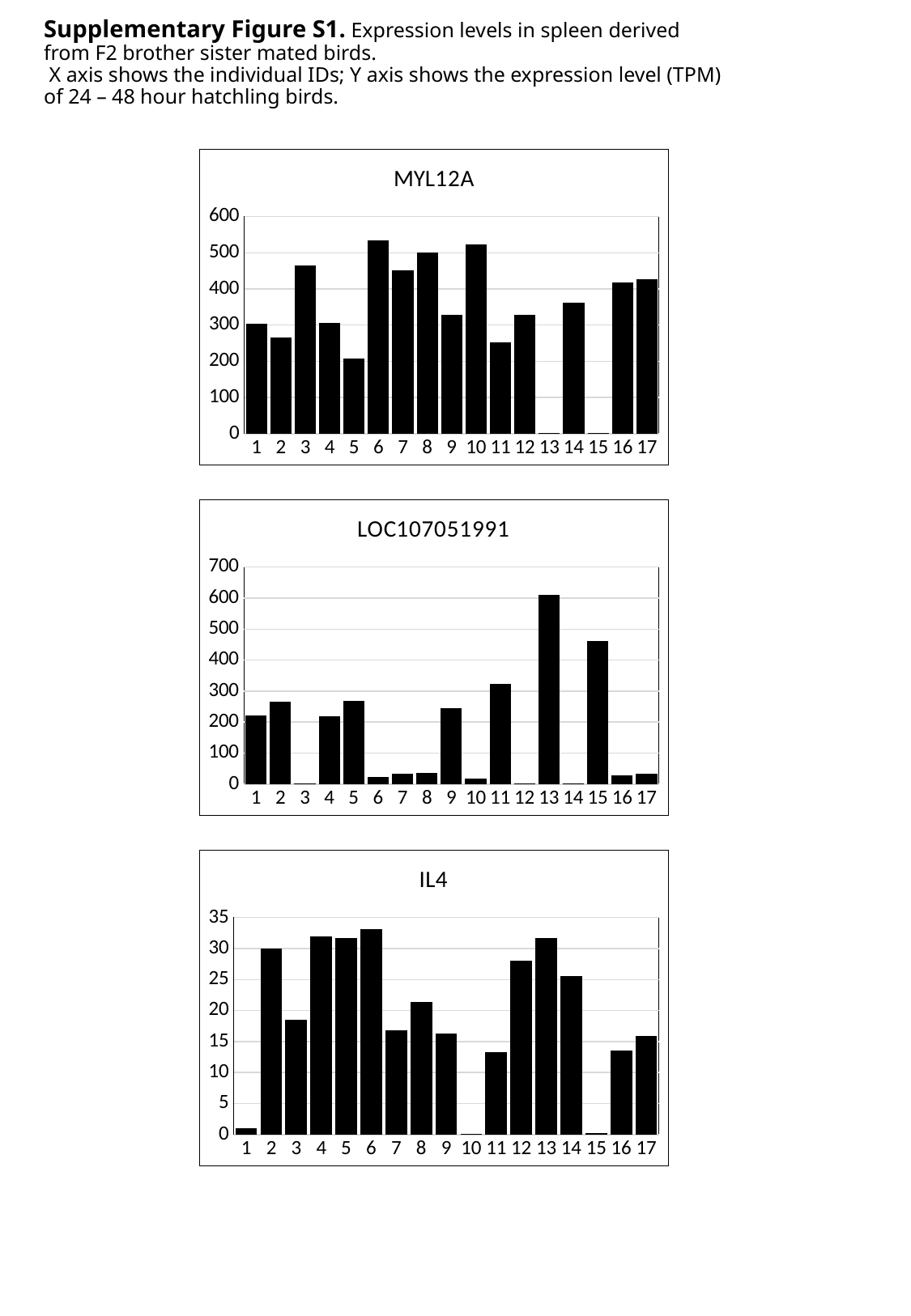

Supplementary Figure S1. Expression levels in spleen derived from F2 brother sister mated birds.
 X axis shows the individual IDs; Y axis shows the expression level (TPM) of 24 – 48 hour hatchling birds.
### Chart: MYL12A
| Category | |
|---|---|
### Chart: LOC107051991
| Category | |
|---|---|
### Chart: IL4
| Category | |
|---|---|

## Slide 2
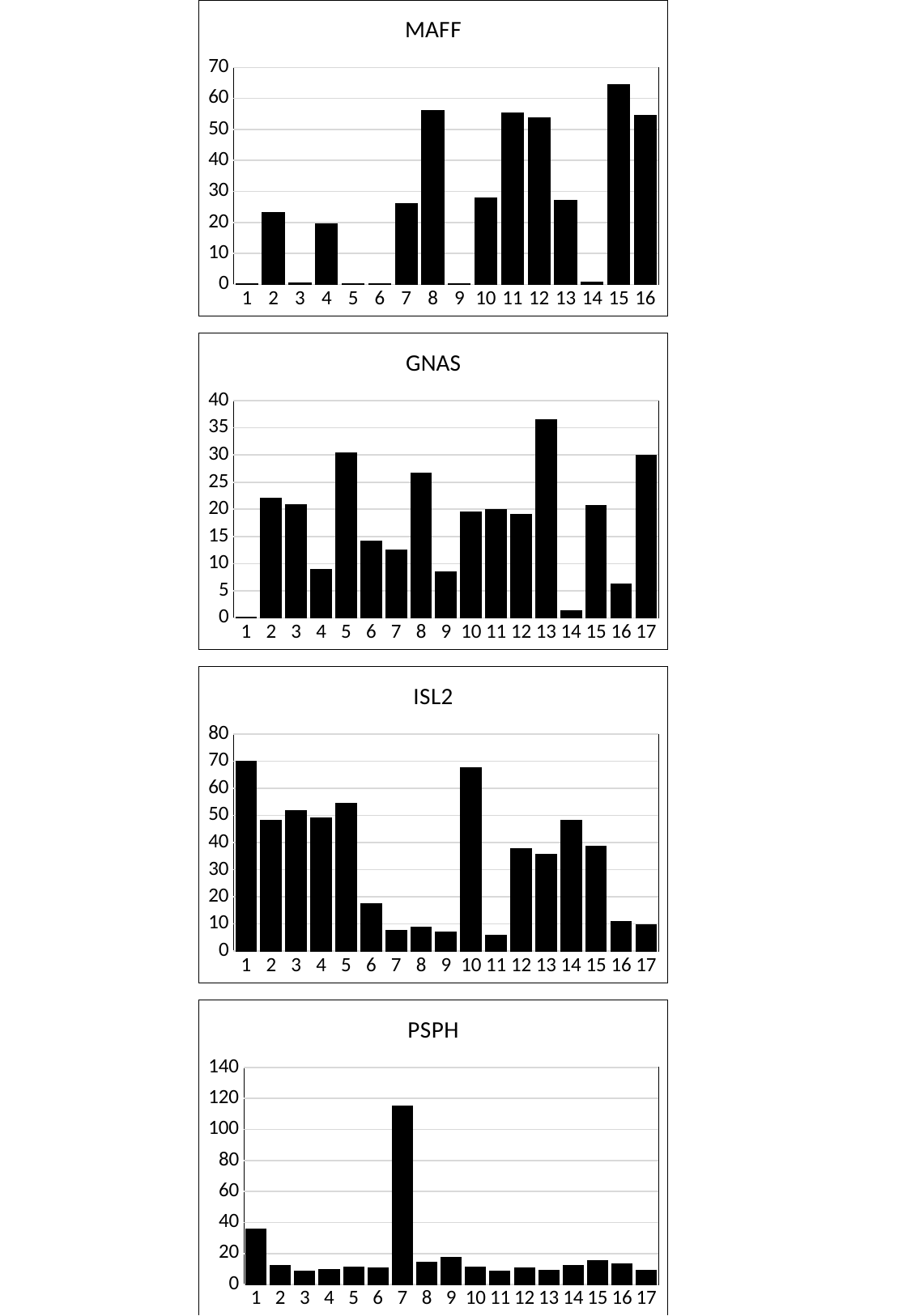

### Chart: MAFF
| Category | |
|---|---|
### Chart: GNAS
| Category | |
|---|---|
### Chart: ISL2
| Category | |
|---|---|
### Chart: PSPH
| Category | |
|---|---|

## Slide 3
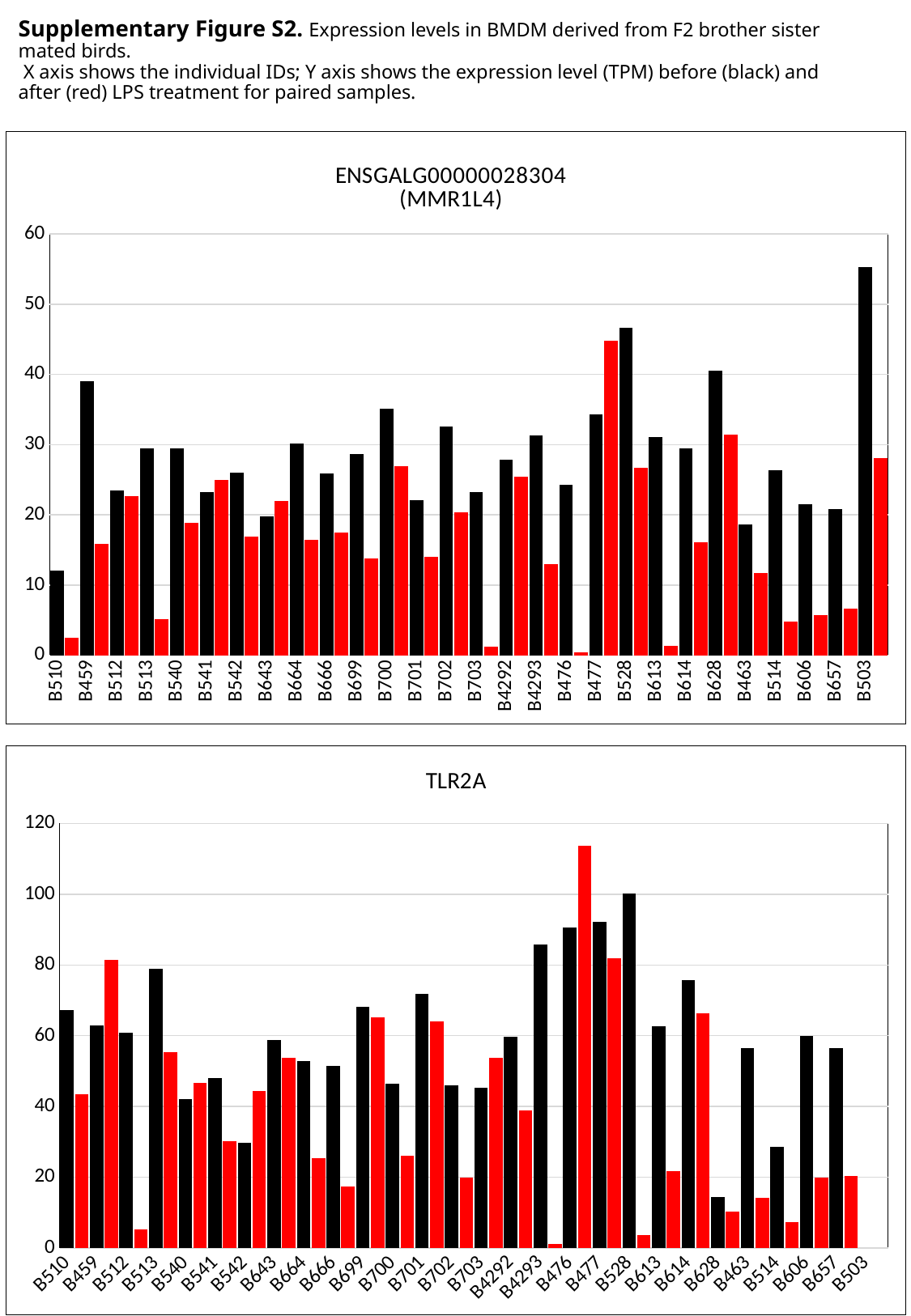

Supplementary Figure S2. Expression levels in BMDM derived from F2 brother sister mated birds.
 X axis shows the individual IDs; Y axis shows the expression level (TPM) before (black) and after (red) LPS treatment for paired samples.
### Chart: ENSGALG00000028304
(MMR1L4)
| Category | |
|---|---|
| B510 | 12.106 |
| B510 | 2.519 |
| B459 | 38.996 |
| B459 | 15.876 |
| B512 | 23.503 |
| B512 | 22.713 |
| B513 | 29.46 |
| B513 | 5.143 |
| B540 | 29.442 |
| B540 | 18.876 |
| B541 | 23.294 |
| B541 | 24.963 |
| B542 | 25.952 |
| B542 | 16.899 |
| B643 | 19.783 |
| B643 | 22.013 |
| B664 | 30.214 |
| B664 | 16.473 |
| B666 | 25.881 |
| B666 | 17.534 |
| B699 | 28.663 |
| B699 | 13.797 |
| B700 | 35.147 |
| B700 | 26.895 |
| B701 | 22.104 |
| B701 | 14.071 |
| B702 | 32.602 |
| B702 | 20.317 |
| B703 | 23.247 |
| B703 | 1.203 |
| B4292 | 27.871 |
| B4292 | 25.468 |
| B4293 | 31.311 |
| B4293 | 12.944 |
| B476 | 24.268 |
| B476 | 0.437 |
| B477 | 34.291 |
| B477 | 44.774 |
| B528 | 46.619 |
| B528 | 26.667 |
| B613 | 31.097 |
| B613 | 1.373 |
| B614 | 29.455 |
| B614 | 16.134 |
| B628 | 40.501 |
| B628 | 31.449 |
| B463 | 18.579 |
| B463 | 11.709 |
| B514 | 26.328 |
| B514 | 4.818 |
| B606 | 21.51 |
| B606 | 5.767 |
| B657 | 20.814 |
| B657 | 6.664 |
| B503 | 55.296 |
| B503 | 28.122 |
[unsupported chart]

## Slide 4
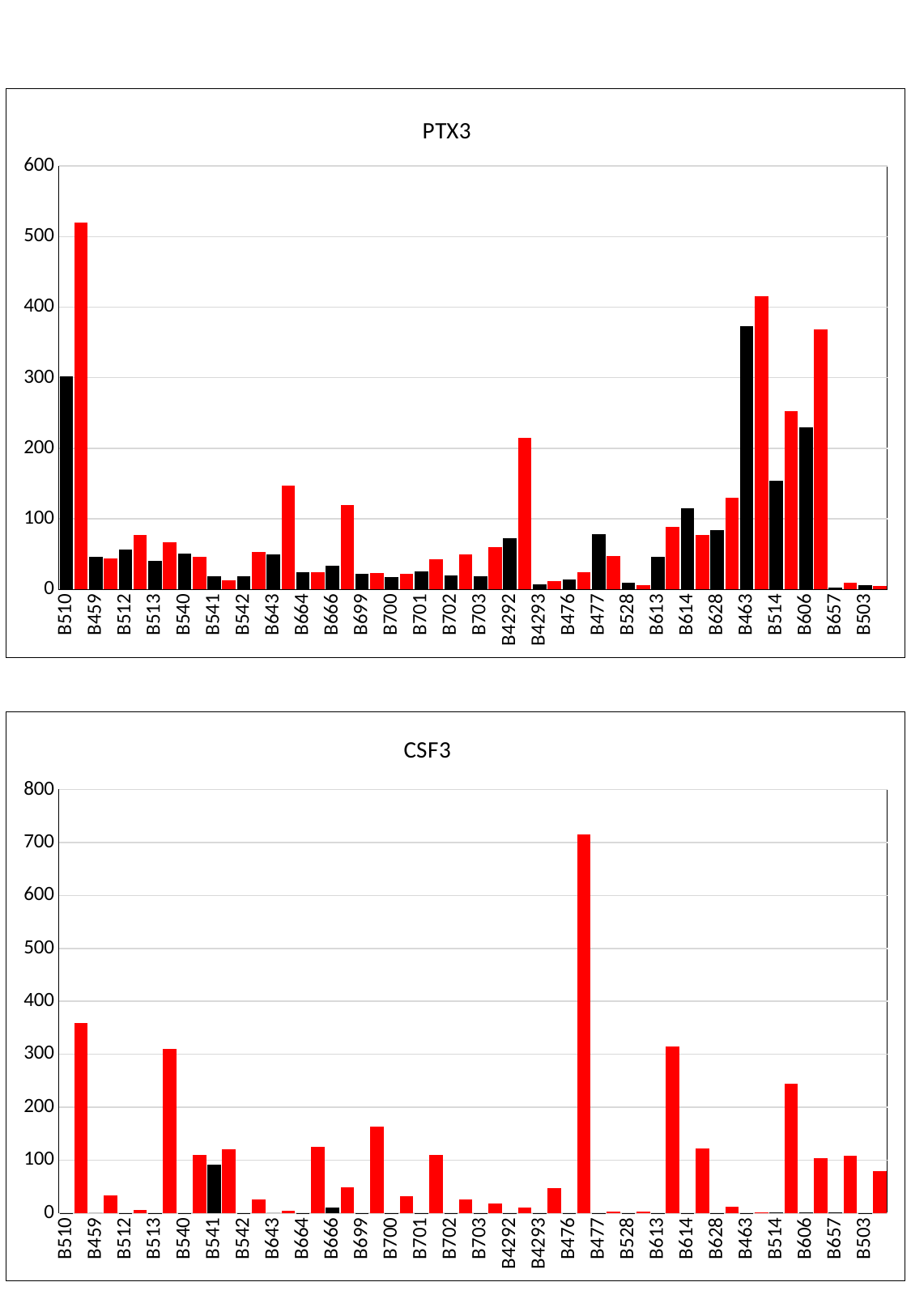

### Chart: PTX3
| Category | |
|---|---|
| B510 | 302.203 |
| B510 | 520.25 |
| B459 | 46.314 |
| B459 | 43.536 |
| B512 | 56.74 |
| B512 | 76.65 |
| B513 | 40.533 |
| B513 | 66.728 |
| B540 | 50.709 |
| B540 | 46.405 |
| B541 | 18.187 |
| B541 | 13.212 |
| B542 | 18.199 |
| B542 | 53.571 |
| B643 | 49.49 |
| B643 | 147.694 |
| B664 | 23.909 |
| B664 | 23.931 |
| B666 | 33.786 |
| B666 | 119.905 |
| B699 | 21.722 |
| B699 | 23.488 |
| B700 | 17.708 |
| B700 | 21.741 |
| B701 | 26.044 |
| B701 | 43.321 |
| B702 | 19.902 |
| B702 | 50.146 |
| B703 | 19.02 |
| B703 | 59.548 |
| B4292 | 72.948 |
| B4292 | 214.504 |
| B4293 | 7.078 |
| B4293 | 12.312 |
| B476 | 14.504 |
| B476 | 24.2 |
| B477 | 77.996 |
| B477 | 46.962 |
| B528 | 9.543 |
| B528 | 5.998 |
| B613 | 45.971 |
| B613 | 88.747 |
| B614 | 115.268 |
| B614 | 77.475 |
| B628 | 83.907 |
| B628 | 129.816 |
| B463 | 373.388 |
| B463 | 415.301 |
| B514 | 153.922 |
| B514 | 252.713 |
| B606 | 229.986 |
| B606 | 368.73 |
| B657 | 2.768 |
| B657 | 9.44 |
| B503 | 6.277 |
| B503 | 5.435 |
### Chart: CSF3
| Category | |
|---|---|
| B510 | 0.331 |
| B510 | 358.469 |
| B459 | 0.0 |
| B459 | 33.426 |
| B512 | 0.132 |
| B512 | 6.312 |
| B513 | 0.163 |
| B513 | 309.87 |
| B540 | 0.258 |
| B540 | 109.406 |
| B541 | 92.15 |
| B541 | 120.834 |
| B542 | 0.085 |
| B542 | 25.955 |
| B643 | 0.0 |
| B643 | 3.585 |
| B664 | 0.312 |
| B664 | 125.603 |
| B666 | 10.165 |
| B666 | 48.374 |
| B699 | 0.202 |
| B699 | 163.01 |
| B700 | 0.126 |
| B700 | 31.75 |
| B701 | 0.157 |
| B701 | 109.606 |
| B702 | 0.128 |
| B702 | 25.571 |
| B703 | 0.058 |
| B703 | 18.635 |
| B4292 | 0.135 |
| B4292 | 10.788 |
| B4293 | 0.214 |
| B4293 | 47.564 |
| B476 | 0.213 |
| B476 | 715.322 |
| B477 | 0.026 |
| B477 | 2.63 |
| B528 | 0.105 |
| B528 | 2.017 |
| B613 | 0.106 |
| B613 | 315.355 |
| B614 | 0.102 |
| B614 | 121.667 |
| B628 | 0.081 |
| B628 | 11.764 |
| B463 | 0.165 |
| B463 | 1.91 |
| B514 | 1.379 |
| B514 | 244.573 |
| B606 | 0.499 |
| B606 | 104.204 |
| B657 | 1.604 |
| B657 | 108.791 |
| B503 | 0.099 |
| B503 | 79.864 |

## Slide 5
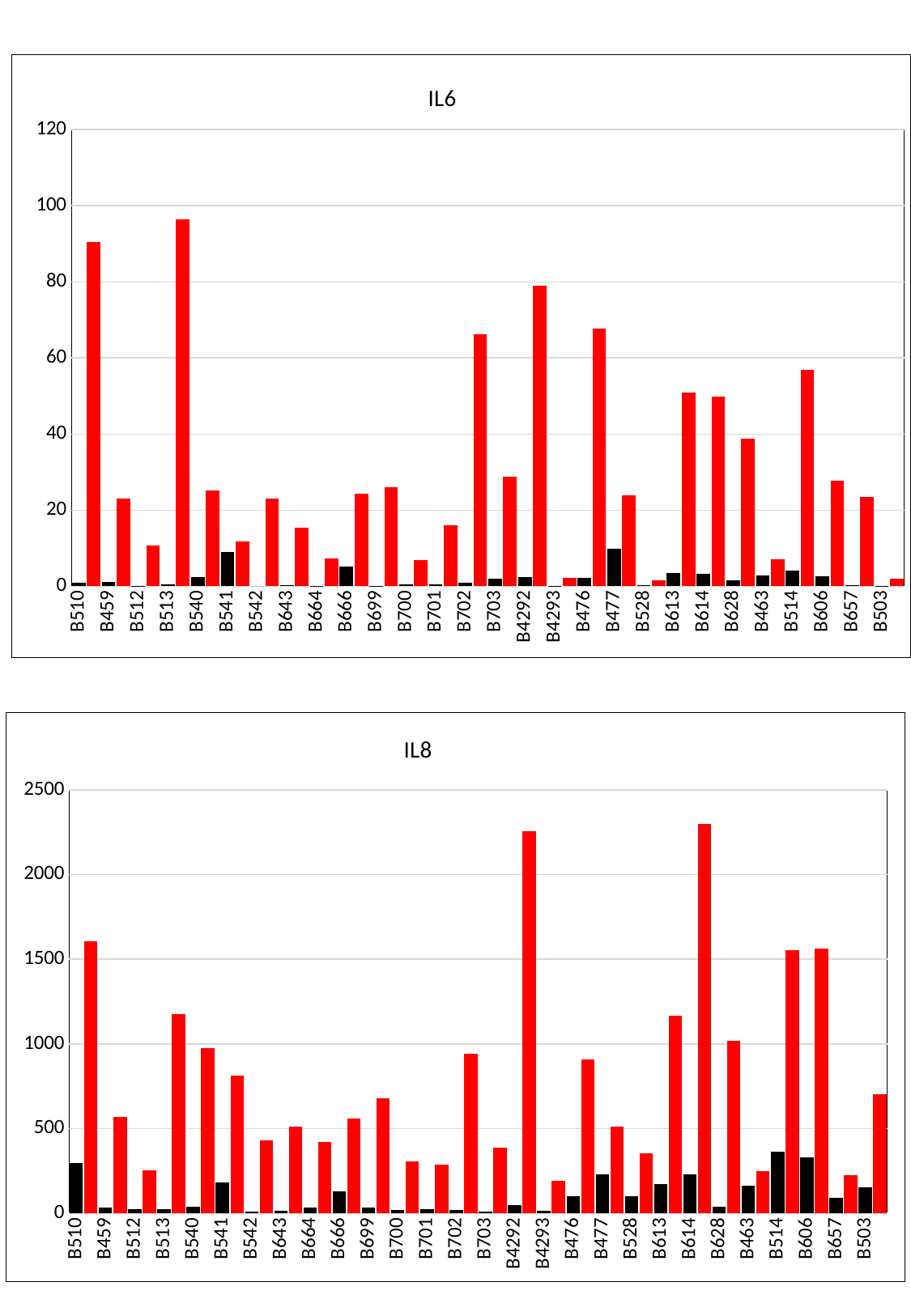

### Chart: IL6
| Category | |
|---|---|
| B510 | 0.864 |
| B510 | 90.505 |
| B459 | 1.057 |
| B459 | 23.055 |
| B512 | 0.139 |
| B512 | 10.684 |
| B513 | 0.513 |
| B513 | 96.524 |
| B540 | 2.365 |
| B540 | 25.143 |
| B541 | 8.994 |
| B541 | 11.825 |
| B542 | 0.0 |
| B542 | 22.984 |
| B643 | 0.225 |
| B643 | 15.387 |
| B664 | 0.142 |
| B664 | 7.352 |
| B666 | 5.217 |
| B666 | 24.326 |
| B699 | 0.067 |
| B699 | 26.008 |
| B700 | 0.432 |
| B700 | 6.95 |
| B701 | 0.609 |
| B701 | 15.989 |
| B702 | 0.983 |
| B702 | 66.139 |
| B703 | 1.92 |
| B703 | 28.886 |
| B4292 | 2.491 |
| B4292 | 79.031 |
| B4293 | 0.048 |
| B4293 | 2.305 |
| B476 | 2.265 |
| B476 | 67.825 |
| B477 | 9.768 |
| B477 | 23.943 |
| B528 | 0.259 |
| B528 | 1.504 |
| B613 | 3.551 |
| B613 | 50.892 |
| B614 | 3.259 |
| B614 | 49.874 |
| B628 | 1.649 |
| B628 | 38.824 |
| B463 | 2.813 |
| B463 | 7.052 |
| B514 | 4.021 |
| B514 | 56.966 |
| B606 | 2.554 |
| B606 | 27.64 |
| B657 | 0.206 |
| B657 | 23.572 |
| B503 | 0.121 |
| B503 | 2.012 |
### Chart: IL8
| Category | |
|---|---|
| B510 | 298.218 |
| B510 | 1607.22 |
| B459 | 35.792 |
| B459 | 567.017 |
| B512 | 24.803 |
| B512 | 253.216 |
| B513 | 23.902 |
| B513 | 1174.71 |
| B540 | 36.141 |
| B540 | 976.405 |
| B541 | 180.691 |
| B541 | 813.022 |
| B542 | 8.833 |
| B542 | 430.081 |
| B643 | 12.136 |
| B643 | 512.454 |
| B664 | 31.703 |
| B664 | 419.67 |
| B666 | 129.743 |
| B666 | 557.042 |
| B699 | 33.845 |
| B699 | 680.646 |
| B700 | 21.268 |
| B700 | 306.358 |
| B701 | 22.901 |
| B701 | 288.049 |
| B702 | 17.812 |
| B702 | 941.178 |
| B703 | 10.222 |
| B703 | 389.625 |
| B4292 | 49.517 |
| B4292 | 2256.39 |
| B4293 | 14.036 |
| B4293 | 192.875 |
| B476 | 101.659 |
| B476 | 908.863 |
| B477 | 231.619 |
| B477 | 510.47 |
| B528 | 98.308 |
| B528 | 355.087 |
| B613 | 170.54 |
| B613 | 1164.17 |
| B614 | 228.531 |
| B614 | 2296.81 |
| B628 | 37.908 |
| B628 | 1019.33 |
| B463 | 160.362 |
| B463 | 248.285 |
| B514 | 363.234 |
| B514 | 1554.75 |
| B606 | 328.236 |
| B606 | 1562.28 |
| B657 | 90.835 |
| B657 | 226.545 |
| B503 | 151.87 |
| B503 | 702.332 |

## Slide 6
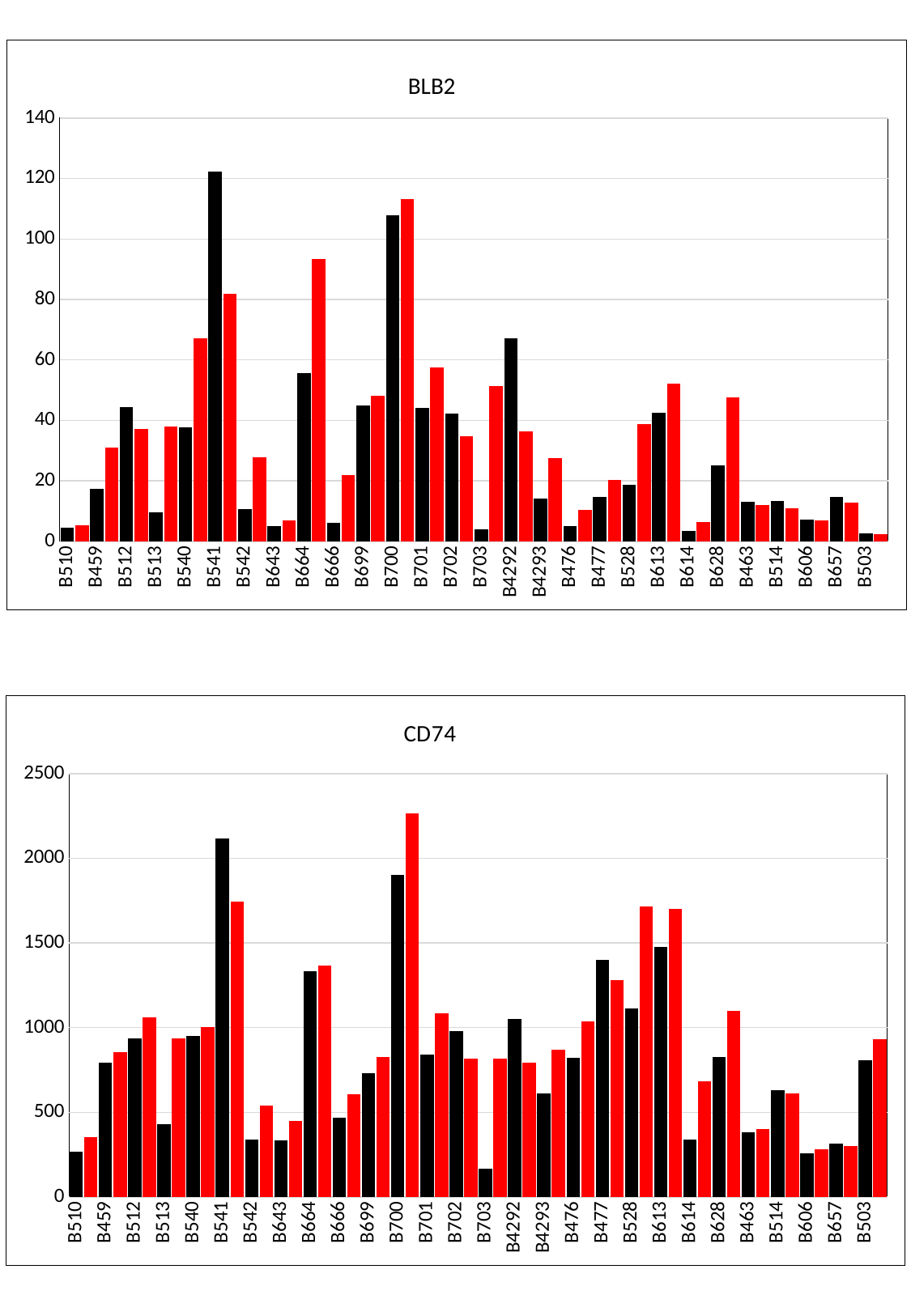

### Chart: BLB2
| Category | |
|---|---|
| B510 | 4.574 |
| B510 | 5.228 |
| B459 | 17.344 |
| B459 | 31.129 |
| B512 | 44.437 |
| B512 | 37.104 |
| B513 | 9.566 |
| B513 | 37.969 |
| B540 | 37.71 |
| B540 | 67.238 |
| B541 | 122.37 |
| B541 | 81.765 |
| B542 | 10.743 |
| B542 | 27.698 |
| B643 | 4.915 |
| B643 | 6.865 |
| B664 | 55.614 |
| B664 | 93.345 |
| B666 | 6.042 |
| B666 | 21.792 |
| B699 | 44.968 |
| B699 | 48.085 |
| B700 | 107.867 |
| B700 | 113.164 |
| B701 | 44.135 |
| B701 | 57.565 |
| B702 | 42.231 |
| B702 | 34.831 |
| B703 | 4.015 |
| B703 | 51.41 |
| B4292 | 67.051 |
| B4292 | 36.472 |
| B4293 | 14.134 |
| B4293 | 27.633 |
| B476 | 4.936 |
| B476 | 10.539 |
| B477 | 14.702 |
| B477 | 20.407 |
| B528 | 18.634 |
| B528 | 38.785 |
| B613 | 42.569 |
| B613 | 52.101 |
| B614 | 3.455 |
| B614 | 6.354 |
| B628 | 25.192 |
| B628 | 47.6 |
| B463 | 13.118 |
| B463 | 11.975 |
| B514 | 13.306 |
| B514 | 10.817 |
| B606 | 7.279 |
| B606 | 6.843 |
| B657 | 14.596 |
| B657 | 12.704 |
| B503 | 2.772 |
| B503 | 2.487 |
### Chart: CD74
| Category | |
|---|---|
| B510 | 269.393 |
| B510 | 352.016 |
| B459 | 793.876 |
| B459 | 857.218 |
| B512 | 936.261 |
| B512 | 1060.558 |
| B513 | 427.473 |
| B513 | 935.679 |
| B540 | 951.359 |
| B540 | 1004.089 |
| B541 | 2115.76 |
| B541 | 1743.06 |
| B542 | 340.828 |
| B542 | 540.143 |
| B643 | 333.651 |
| B643 | 446.877 |
| B664 | 1333.293 |
| B664 | 1364.758 |
| B666 | 468.106 |
| B666 | 607.734 |
| B699 | 732.337 |
| B699 | 823.84 |
| B700 | 1902.295 |
| B700 | 2263.747 |
| B701 | 842.553 |
| B701 | 1083.815 |
| B702 | 977.182 |
| B702 | 817.179 |
| B703 | 167.991 |
| B703 | 815.824 |
| B4292 | 1049.758 |
| B4292 | 790.559 |
| B4293 | 609.789 |
| B4293 | 868.823 |
| B476 | 823.405 |
| B476 | 1035.814 |
| B477 | 1399.223 |
| B477 | 1282.751 |
| B528 | 1111.498 |
| B528 | 1717.629 |
| B613 | 1478.21 |
| B613 | 1700.005 |
| B614 | 338.902 |
| B614 | 681.251 |
| B628 | 825.802 |
| B628 | 1098.547 |
| B463 | 382.489 |
| B463 | 401.851 |
| B514 | 632.547 |
| B514 | 610.51 |
| B606 | 257.016 |
| B606 | 279.523 |
| B657 | 313.474 |
| B657 | 300.208 |
| B503 | 807.772 |
| B503 | 930.835 |
